# Supplementary material for: Physiological and Transcriptional Responses of Apocynum venetum to Salt Stress at the Seed Germination Stage
Source: Int J Mol Sci. 2023 Feb 11;24(4):3623. doi: 10.3390/ijms24043623 (PMC9966927; doi:10.3390/ijms24043623)
Supplement: Supplementary file 1 [file ijms-24-03623-s001.zip › ijms-2204216-supplementary.pdf]

Supplementary Materials

Figure supplementary legends

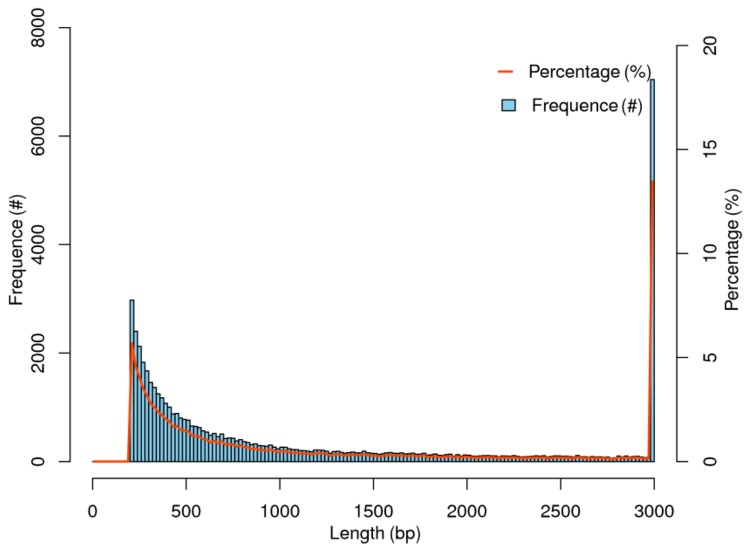

Figure S1. Length distribution of assembled unigenes.

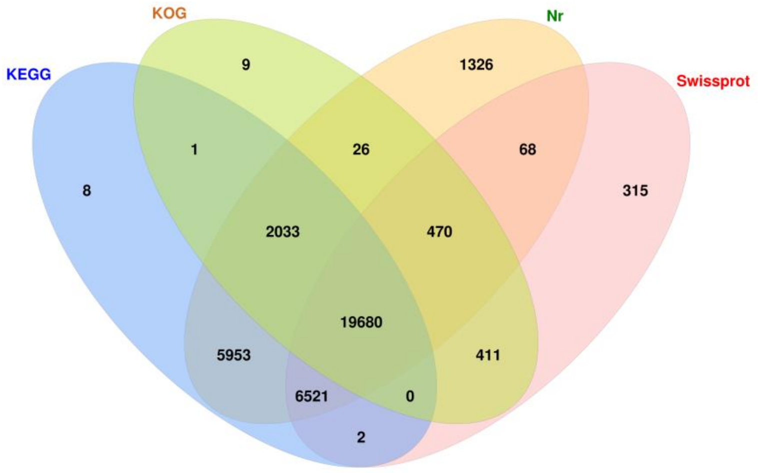

Figure S2. Basic annotation for all unigenes in *A. venetum* on KEGG, KOG, Nr, and Swissprot databases.

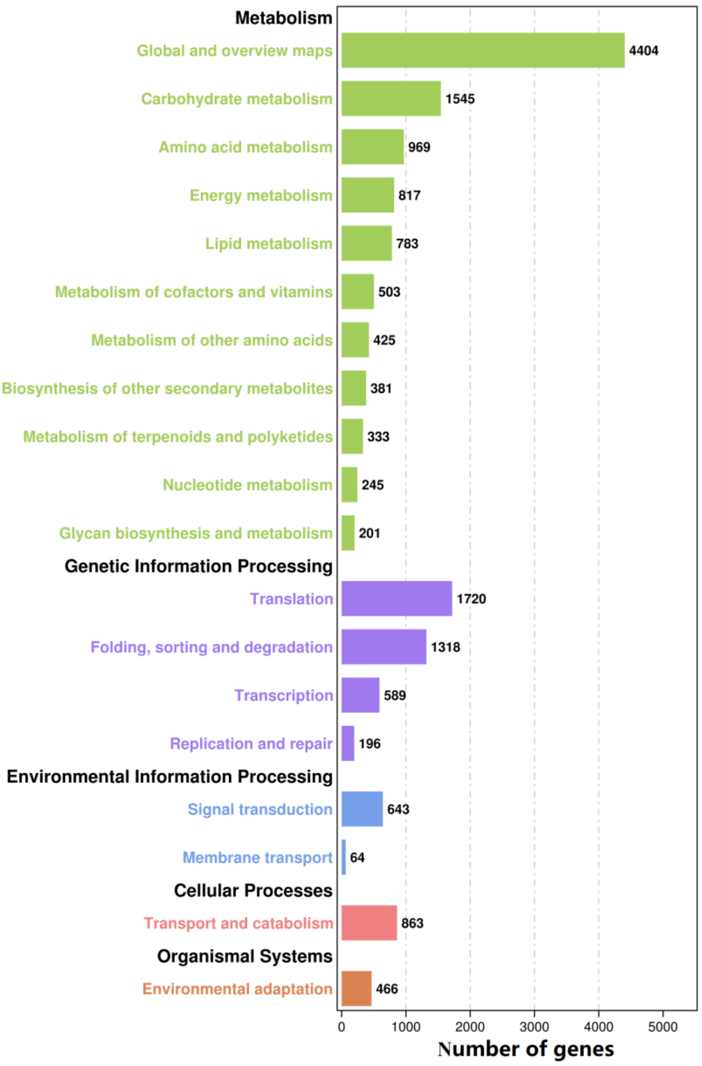

Figure S3. KEGG enrichment of DEGs at NaCl (300 mmol/L) vs. CK.

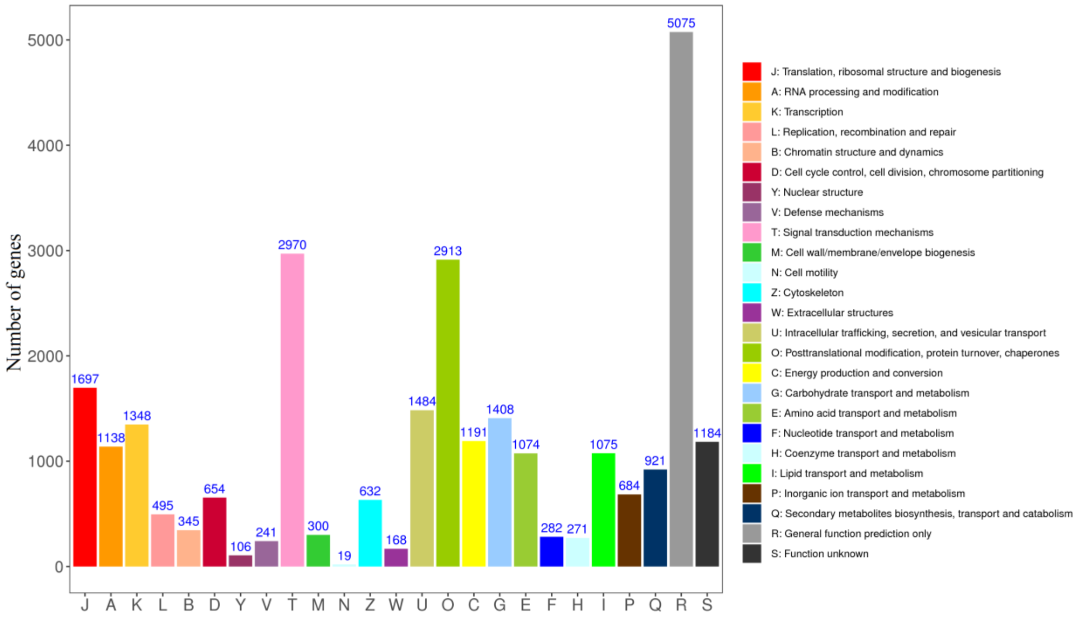

Figure S4. Distribution of unigenes in the transcriptome with KOG functional classification.

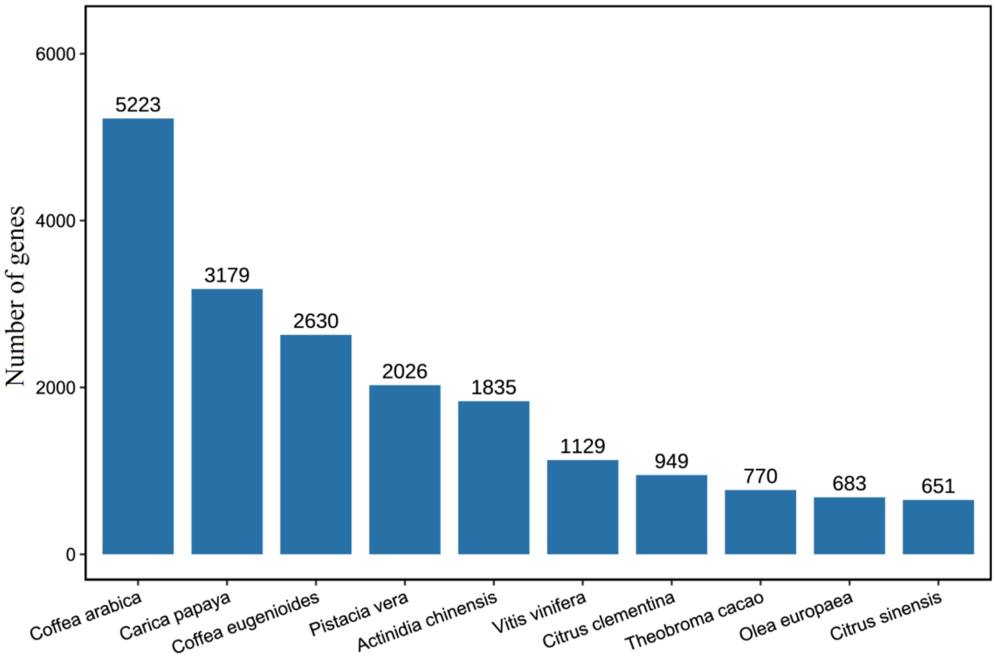

Figure S5. Annotation of unigenes on NR database and distribution of the top 10 species.

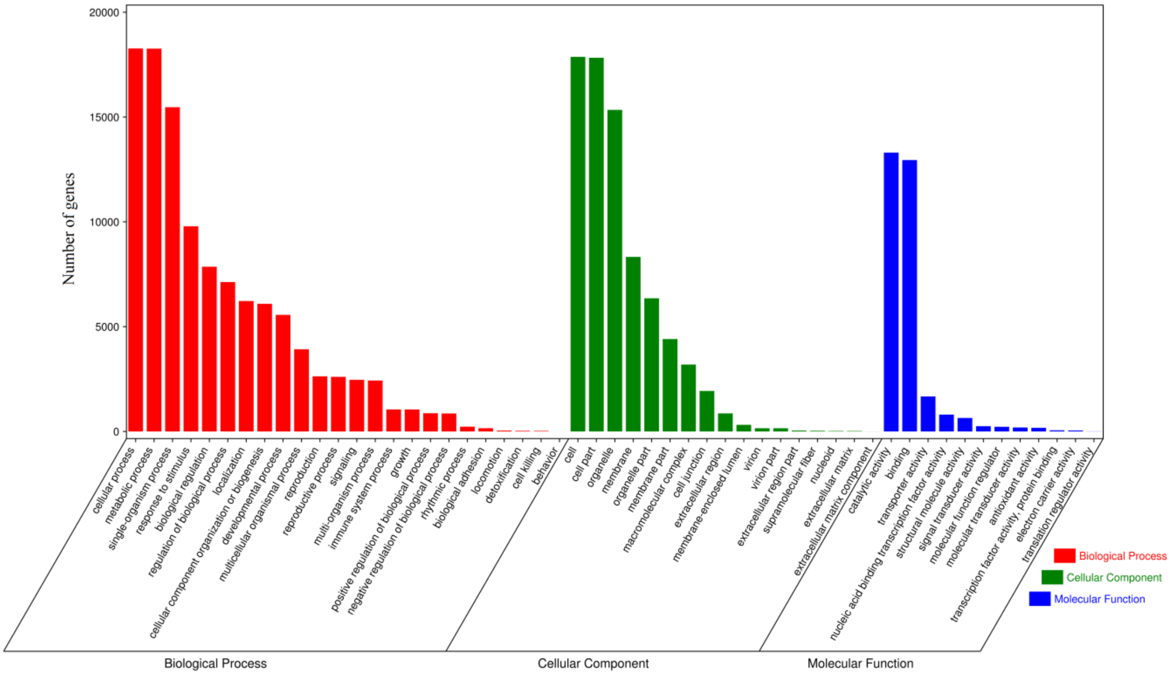

Figure S6. Annotation of unigenes on GO database and biological classification.

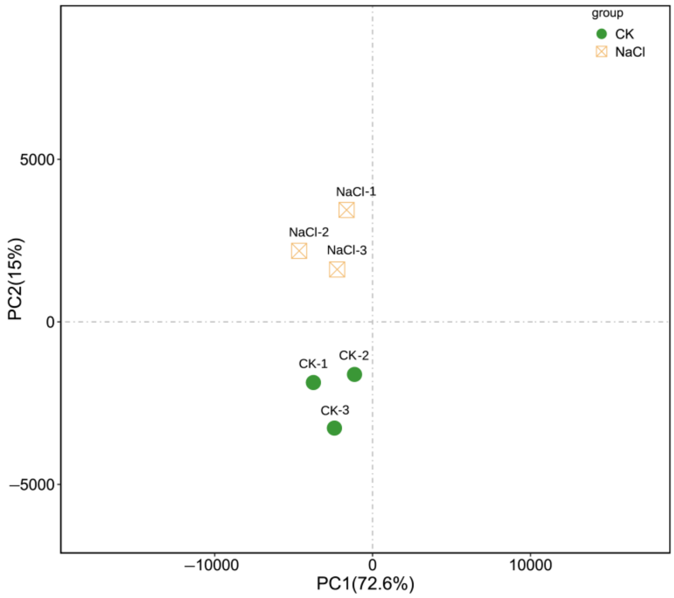

Figure S7. Principal component analysis (PCA) of CK and NaCl (300 mmol/L).

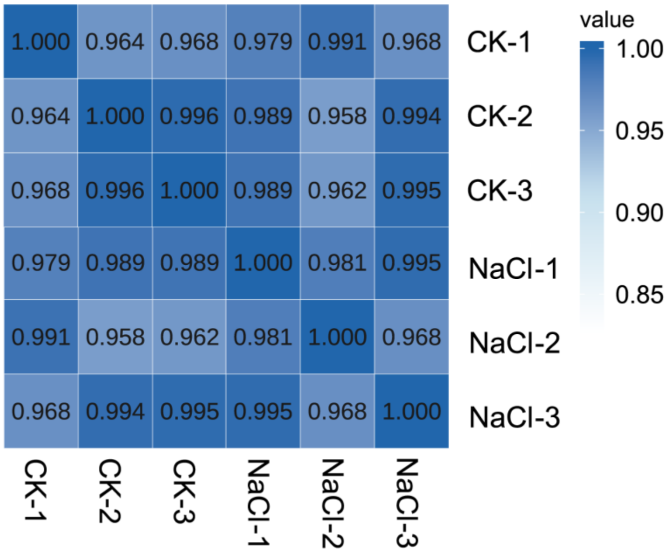

Figure S8. Pearson correlation analysis of CK and NaCl (300 mmol/L).

Table supplementary legends

Table S1. One hundred and twenty-three genes associated with other stress response at NaCl (300 mmol/L) vs. CK.

Gene name	SwissProt ID	Protein name	log_2FC (NaCl vs. CK)
**Response to heat and cold**			
HSP18.5-C	P05478	18.5 kDa class I heat shock protein	-1.40
HSPD1	P10809	60 kDa heat shock protein, mitochondrial	8.44
COR413PM2	Q9SVL6	Cold-regulated 413 plasma membrane protein 2	3.85
HSP70-4	Q9LHA8	Heat shock 70 kDa protein 4	9.70
HSP70-14	Q9S7C0	Heat shock 70 kDa protein 14	4.23
HSC-2	P27322	Heat shock cognate 70 kDa protein 2	4.26
HSPA8	A2Q0Z1	Heat shock cognate 71 kDa protein	3.68
HSC80	P36181	Heat shock cognate protein 80	1.30
HSP81-2	Q69QQ6	Heat shock protein 81-2	-9.91
HSP83A	P51819	Heat shock protein 83	-1.02
HSP90-5	Q9SIF2	Heat shock protein 90-5, chloroplastic	3.80
HSP90-6	F4JFN3	Heat shock protein 90-6, mitochondrial	8.42
HSP90AB1	P08238	Heat shock protein HSP 90-beta	5.70
HSFA2B	Q6VBB2	Heat stress transcription factor A-2b	1.86
HSFB3	O22230	Heat stress transcription factor B-3	2.73
HOP2	Q5XEP2	Hsp70-Hsp90 organizing protein 2	4.31
LTI65	Q04980	Low-temperature-induced 65 kDa protein	3.24
HSP70	Q02028	Stromal 70 kDa heat shock-related protein, chloroplastic	4.17
**Others**			
At1g06620	Q84MB3	1-aminocyclopropane-1-carboxylate oxidase homolog 1	-2.37
At1g06650	Q8H1S4	1-aminocyclopropane-1-carboxylate oxidase homolog 3	3.03
At1g03400	Q94A78	1-aminocyclopropane-1-carboxylate oxidase homolog 4	-1.91
At1g43910	Q9LP11	AAA-ATPase At1g43910	8.89
ABRA	P11140	Abrin-a	8.82
AAG	Q9M6E9	Agglutinin-1	5.47
ALDH2B4	Q9SU63	Aldehyde dehydrogenase family 2 member B4, mitochondrial	4.12
ALD1	Q9ZQI7	Aminotransferase ALD1, chloroplastic	8.89
PRB1	P11670	Basic form of pathogenesis-related protein 1	-1.06
FOX2	Q9FZC5	Berberine bridge enzyme-like 4	-4.01
At1g30700	Q9SA85	Berberine bridge enzyme-like 8	-3.38
MEE23	O64743	Berberine bridge enzyme-like 15	-1.04
CATHB2	Q93VC9	Cathepsin B-like protease 2	4.83
CBSX2	Q9C5D0	CBS domain-containing protein CBSX2, chloroplastic	8.80
DJA6	Q0JB88	Chaperone protein dnaJ A6	4.00
Chga	P26339	Chromogranin-A	6.05
CRY1	Q43125	Cryptochrome-1	4.63
XCP1	O65493	Cysteine protease XCP1	-10.84
XCP2	Q9LM66	Cysteine protease XCP2	4.98
CYTI	Q06445	Cysteine proteinase inhibitor	10.19
CYS6	Q8H0X6	Cysteine proteinase inhibitor 6	9.09
CYS7	Q8LC76	Cysteine proteinase inhibitor 7	9.96
DEF	A3FPF2	Defensin-like protein	4.81
DHN1	P12950	Dehydrin DHN1	3.25
LPD1	Q9M5K3	Dihydrolipoyl dehydrogenase 1, mitochondrial	4.45
CjBAp12	Q9ZP41	EG45-like domain containing protein	11.34
GUN	P22503	Endoglucanase	-6.57
CHI1	Q39799	Endochitinase 1	8.62
ENDO4	F4JJL0	Endonuclease 4	3.49
KOR	Q38890	Endoglucanase 25	2.96
EP3	Q9M2U5	Endochitinase EP3	7.32
At1g78830	Q9ZVA2	EP1-like glycoprotein 2	-2.05
At1g78850	Q9ZVA4H	EP1-like glycoprotein 3	3.32
ephA	I6YGS0	Epoxide hydrolase A	-4.41
FLZ14	Q8GYX2	FCS-Like Zinc finger 14	3.56
hmp	Q7WHW5	Flavohemoprotein	-9.78
At3g05950	Q9SFF9	Germin-like protein subfamily 1 member 7	-3.22
GLP10	Q9M263	Germin-like protein subfamily 2 member 4	4.60
GLYR1	Q9LSV0	Glyoxylate/succinic semialdehyde reductase 1	4.15
GLYR2	F4I907	Glyoxylate/succinic semialdehyde reductase 2, chloroplastic	4.96

HEL	P43082	Hevein-like preproprotein	-2.27
RCD1	Q8RY59	Inactive poly [ADP-ribose] polymerase RCD1	5.11
PPF-1	Q9FY06	Inner membrane protein PPF-1, chloroplastic	3.41
KTI5	Q9LMU2	Kunitz trypsin inhibitor 5	10.79
Bp10	Q00624	L-ascorbate oxidase homolog	3.11
LRR1	Q9FPJ5	Leucine-rich repeat protein 1	4.97
LRR2	Q6NQP4	Leucine-rich repeat protein 2	5.61
MIF	P14174	Macrophage migration inhibitory factor	9.74
PRU1	O50001	Major allergen Pru ar 1	7.56
Mup1	P11588	Major urinary protein 1	-12.22
MLO1	O49621	MLO-like protein 1	1.96
MLP28	Q9SSK9	MLP-like protein 28	4.00
MLP328	Q9ZVF3	MLP-like protein 328	3.99
MLP423	Q93VR4	MLP-like protein 423	2.71
HSPRO2	O04203	Nematode resistance protein-like HSPRO2	4.24
NHL1	Q9SRN0	NDR1/HIN1-like protein 1	− 2.84
NHL6	Q8LD98	NDR1/HIN1-like protein 6	3.32
NHL10	Q9SJ52	NDR1/HIN1-like protein 10	4.38
NHL13	Q9ZVD2	NDR1/HIN1-like protein 13	− 1.15
AGP31	Q9FZA2	Non-classical arabinogalactan protein 31	4.70
NUDT8	Q8L7W2	Nudix hydrolase 8	-1.08
NUDT17	Q9ZU95	Nudix hydrolase 17, mitochondrial	4.69
OSM34	P50700	Osmotin-like protein OSM34	4.35
AAE3	Q9SMT7	Oxalate--CoA ligase	9.37
PCO2	Q8LGJ5	Plant cysteine oxidase 2	-4.29
PLAT2	Q9SIE7	PLAT domain-containing protein 2	3.99
CEP14	Q52K95	Precursor of CEP14	-1.23
GIP2	P0DO21	Probable aspartic proteinase GIP2	7.13
CSA	Q06652	Probable phospholipid hydroperoxide glutathione peroxidase	4.37
HEV1	P02877	Pro-hevein	12.11
ASPG1	Q9LS40	Protein ASPARTIC PROTEASE IN GUARD CELL 1	3.73
DJ1D	Q9M8R4	Protein DJ-1 homolog D	4.17
EXL2	Q9FE06	Protein EXORDIUM-like 2	3.53
EXL3	Q9FHM9	Protein EXORDIUM-like 3	4.44
EXL5	Q9SII5	Protein EXORDIUM-like 5	3.22
JOKA2	M1BJF6	Protein JOKA2	5.93
SRC2	O04023	Protein SRC2 homolog	4.54
At5g40382	Q9FNE0	Putative cytochrome c oxidase subunit 5C-4	1.07
RGA3	Q7XA40	Putative disease resistance protein RGA3	1.67
PDC2	Q9FFT4	Pyruvate decarboxylase 2	-2.18
rcf2	Q9P3B2	Respiratory supercomplex factor 2 homolog C1565.01	-9.04
PO21	Q03278	Retrovirus-related Pol polyprotein from type-1 retrotransposable element R2	4.32
RggA	Q9SQ56	RGG repeats nuclear RNA binding protein A	3.84
RGGB	O23593	RGG repeats nuclear RNA binding protein B	4.26
DFC	Q9LX15	Protein DOWNSTREAM OF FLC	5.05
SAG20	Q94AK6	Senescence associated gene 20	-2.64
SERINC3	Q5R533	Serine incorporator 3	5.25
SN1	Q948Z4	Snakin-1	-1.01
Sst	P60041	Somatostatin	5.34
At5g22580	Q9FK81	Stress-response A/B barrel domain-containing protein At5g22580	10.14
ICIS	P08820	Subtilisin inhibitor	10.17
TLP	P83958	Thaumatin-like protein	-2.17
At5g16400	Q9XFH9	Thioredoxin F2, chloroplastic	5.63
TRXH	Q43636	Thioredoxin H-type	3.37
At3g15360	Q9SEU6	Thioredoxin M4, chloroplastic	3.61
ATHX	Q8LD49	Thioredoxin X, chloroplastic	9.30
At1g08570	O64654	Thioredoxin-like 1-1, chloroplastic	3.90
CDSP32	Q9SGS4	Thioredoxin-like protein CDSP32, chloroplastic	2.89
TL17	P81760	Thylakoid lumenal 17.4 kDa protein, chloroplastic	5.14
CLEB3J9	Q9THX6	Thylakoid lumenal 29 kDa protein, chloroplastic	5.61
PHOS34	Q8L4N1	Universal stress protein PHOS34	4.18
At3g17800	Q9LVJ0	UV-B-induced protein At3g17800, chloroplastic	4.21
WIN2	P09762	Wound-induced protein WIN2	-1.40
SAP4	Q9SJM6	Zinc finger A20 and AN1 domain-containing stress-associated protein 4	5.06
SAP8	A2YEZ6	Zinc finger A20 and AN1 domain-containing stress-associated protein 8	4.67

Table S2. Two hundred and eight genes associated with other primary metabolism at NaCl (300 mmol/L) vs. CK.

Gene name	SwissProt ID	Protein name	log_2FC (NaCl vs. CK)
**Carbohydrate metabolism**			
SMT2	Q39227	24-methylenesterol C-methyltransferase 2	3.04
HMG1	P14891	3-hydroxy-3-methylglutaryl-coenzyme A reductase 1	3.84
ISPG	F4K0E8	4-hydroxy-3-methylbut-2-en-1-yl diphosphate synthase (ferredoxin)	0.72
ISPH	Q94B35	4-hydroxy-3-methylbut-2-enyl diphosphate reductase	4.84
ACO1	Q42560	Aconitate hydratase 1	4.31
ACOC	P49608	Aconitate hydratase, cytoplasmic	3.48
ACA7	Q8L817	Alpha carbonic anhydrase 7	-3.10
PHSL1	P04045	Alpha-1,4 glucan phosphorylase L-1 isozyme	4.75
STP-1	P53535	Alpha-1,4 glucan phosphorylase L-2 isozyme	4.56
AMY1.1	P17859	Alpha-amylase	-3.91
PHSH	P53537	Alpha-glucan phosphorylase, H isozyme	3.92
AGL2	Q9F234	Alpha-glucosidase 2	8.62
BCA4	Q94CE4	Beta carbonic anhydrase 4	3.44
BMY1	O64407	Beta-amylase	5.83
BXL1	Q9FGY1	Beta-D-xylosidase 1	8.20
BFRUCT1	Q43089	Beta-fructofuranosidase, cell wall isozyme	9.83
BGAL	P48980	Beta-galactosidase	4.25
BGAL3	Q9SCV9	Beta-galactosidase 3	3.94
CP12-2	Q9LZP9	Calvin cycle protein CP12-2, chloroplastic	4.10
Os05g0361200	Q0DIV0	Ferrochelatase-2, chloroplastic	3.30
FTHS	P28723	Formate--tetrahydrofolate ligase	4.79
E13A	Q03773	Glucan endo-1,3-beta-glucosidase	-2.25
At4g34480	Q9M069	Glucan endo-1,3-beta-glucosidase 7	4.20
HGN1	P52407	Glucan endo-1,3-beta-glucosidase, basic vacuolar isoform	7.78
CHLY	P23472	Hevamine-A	-5.25
AS	Q9AR73	Hydroquinone glucosyltransferase	8.59
BAM9	Q8VYW2	Inactive beta-amylase 9	4.94
Ins2	P01326	Insulin-2	8.50
IDH5	Q945K7	Isocitrate dehydrogenase [NAD] catalytic subunit 5, mitochondrial	4.66
IDHC	P50218	Isocitrate dehydrogenase [NADP]	4.24
ACEA	P17069	Isocitrate lyase	4.92
ARA1	O23461	L-arabinokinase	3.81
LDHB	P07195	L-lactate dehydrogenase B chain	9.22
MD1	Q9FSF0	Malate dehydrogenase	3.65
MDH1	O48902	Malate dehydrogenase [NADP], chloroplastic	4.38
MDHG	P19446	Malate dehydrogenase, glyoxysomal	4.11
MMDH	P17783	Malate dehydrogenase, mitochondrial	3.60
MASY	P17815	Malate synthase, glyoxysomal	4.40
GANAB	Q14697	Neutral alpha-glucosidase AB	8.37
NIA	P17569	Nitrate reductase [NADH]	3.39
Gba2	Q69ZF3	Non-lysosomal glucosylceramidase	3.57
PPCC	Q9FV65	Phosphoenolpyruvate carboxylase 2	3.73
PPC4	Q8GVE8	Phosphoenolpyruvate carboxylase 4	3.56
At1g56190	P50318	Phosphoglycerate kinase 2, chloroplastic	3.77
PGKY	Q42962	Phosphoglycerate kinase, cytosolic	3.16
At5g64460	Q9FGF0	Phosphoglycerate mutase-like protein 1	4.80
PDAT1	Q9FNA9	Phospholipid:diacylglycerol acyltransferase 1	4.57
algC	Q88BD4	Phosphomannomutase/phosphoglucomutase	2.99
KPPR	P27774	Phosphoribulokinase, chloroplastic	3.59
At1g48100	Q949Z1	Polygalacturonase At1g48100	1.62
At5g22620	Q9FNJ9	Probable 2-carboxy-D-arabinitol-1-phosphatase	3.03
OsI_031067	A2Z3C4	Probable 6-phosphogluconolactonase 4, chloroplastic	8.78
CXE7	Q9ZQ91	Probable carboxylesterase 7	-3.85
RFS2	Q94A08	Probable galactinol--sucrose galactosyltransferase 2	8.81
RFS6	Q8RX87	Probable galactinol--sucrose galactosyltransferase 6	3.07
GSVIVT00026920001	A7PZL3	Probable polygalacturonase	4.74
RPI3	Q9S726	Probable ribose-5-phosphate isomerase 3, chloroplastic	3.99
BGLU41	Q9FIU7	Putative beta-glucosidase 41	-1.62
AAPC	Q40784	Putative glucose-6-phosphate 1-epimerase	5.47
pdcA	Q4WXX9	Pyruvate decarboxylase	-9.54
At3g55800	P46283	Sedoheptulose-1,7-bisphosphatase, chloroplastic	3.66
SHM4	O23254	Serine hydroxymethyltransferase 4	3.92

GLYM	P50433	Serine hydroxymethyltransferase, mitochondrial	3.70
SDH	Q9FJ95	Sorbitol dehydrogenase	9.41
STS1	Q93XK2	Stachyose synthase	1.15
N/A	Q6DQL1	Succinate--CoA ligase [ADP-forming] subunit alpha-2	5.29
TALDO1	P37837	Transaldolase	4.75
TKT	P29401	Transketolase	4.81
TKTC	Q43848	Transketolase, chloroplastic	3.48
GAE1	Q9M0B6	UDP-glucuronate 4-epimerase 1	3.57
GAE6	Q9LIS3	UDP-glucuronate 4-epimerase 6	3.25
UXS5	Q9SN95	UDP-glucuronic acid decarboxylase 5	3.70
XYLA	Q9FKK7	Xylose isomerase	-2.81
**Glycolytic**			
PMGI	P35493	2,3-bisphosphoglycerate-independent phosphoglycerate mutase	3.72
ogdh	Q54JE4	2-oxoglutarate dehydrogenase, mitochondrial	3.14
ENOA	P06733	Alpha-enolase	4.14
PFKP	Q01813	ATP-dependent 6-phosphofructokinase, platelet type	8.61
ENO2	P25696	Bifunctional enolase 2/transcriptional activator	9.09
ENO	P42896	Enolase	4.13
ENO1	Q9LEJ0	Enolase 1	-1.86
GAPDH	P04406	Glyceraldehyde-3-phosphate dehydrogenase	4.84
PGK1	P00558	Phosphoglycerate kinase 1	9.42
PGAM1	P18669	Phosphoglycerate mutase 1	4.04
ODPA	P52902	Pyruvate dehydrogenase E1 component subunit alpha	4.43
PDH-E1 ALPHA	O24457	Pyruvate dehydrogenase E1 component subunit alpha-3	3.23
Os08g0536000	Q6Z1G7	Pyruvate dehydrogenase E1 component subunit beta-1	4.92
OsI_35105	B8BJ39	Pyruvate kinase 1, cytosolic	4.51
Pkm	P52480	Pyruvate kinase PKM	-1.98
KPYC	P22200	Pyruvate kinase, cytosolic isozyme	3.77
TPI1	P60174	Triosephosphate isomerase	3.25
TPIC	P48496	Triosephosphate isomerase, chloroplastic	3.17
TPIP1	P48495	Triosephosphate isomerase, cytosolic	3.33
**Amino metaolism**			
IPMS2	Q9C550	2-isopropylmalate synthase 2, chloroplastic	8.62
IIL1	Q94AR8	3-isopropylmalate dehydratase large subunit, chloroplastic	5.36
At2g45300	P05466	3-phosphoshikimate 1-carboxyvinyltransferase, chloroplastic	3.34
HPD	P93836	4-hydroxyphenylpyruvate dioxygenase	1.05
DHDPS2	Q9FVC8	4-hydroxy-tetrahydrodipicolinate synthase 2, chloroplastic	4.38
MS1	O50008	5-methyltetrahydropteroyltriglutamate--homocysteine methyltransferase 1	3.54
ILVB3	P27819	Acetolactate synthase 3, chloroplastic	4.52
AG118	O04866	Acetylornithine aminotransferase, mitochondrial	8.97
VIT_05s0020g04080	F6HDT7	Acireductone dioxygenase 2	3.92
ACR12	Q9LZ23	ACT domain-containing protein ACR12	9.35
SAHH	P50246	Adenosylhomocysteinase	3.59
AIH	Q8GWW7	Agmatine deiminase	3.89
GDCST	O65396	Aminomethyltransferase, mitochondrial	4.28
ASA1	P32068	Anthranilate synthase alpha subunit 1, chloroplastic	8.69
SPE1	Q39827	Arginine decarboxylase	3.19
AS	O24661	Asparagine synthetase [glutamine-hydrolyzing]	4.49
ASP3	P46644	Aspartate aminotransferase 3, chloroplastic	4.27
ASP5	P46248	Aspartate aminotransferase, chloroplastic	8.32
AKHSDH2	O81852	Bifunctional aspartokinase/homoserine dehydrogenase 2, chloroplastic	2.56
PCAS-1	Q76MX2	Bifunctional L-3-cyanoalanine synthase/cysteine synthase 1	3.95
ctps	Q54V77	CTP synthase	4.29
CGS1	P55217	Cystathionine gamma-synthase 1, chloroplastic	4.38
CYSK	Q43317	Cysteine synthase	4.14
CYSKP	O81155	Cysteine synthase, chloroplastic/chromoplastic	3.72
HEMB1	Q9SFH9	Delta-aminolevulinic acid dehydratase 1, chloroplastic	3.96
LYSA1	Q949X7	Diaminopimelate decarboxylase 1, chloroplastic	3.90
DAPF	Q9LFG2	Diaminopimelate epimerase, chloroplastic	3.76
PYD1	Q9LVI9	Dihydropyrimidine dehydrogenase (NADP(+)), chloroplastic	3.26
PECT1	Q9ZVI9	Ethanolamine-phosphate cytidylyltransferase	5.18
GAD	Q07346	Glutamate decarboxylase	5.58
GAD5	Q9LSH2	Glutamate decarboxylase 5	4.05
EMB2360	P42770	Glutathione reductase, chloroplastic	3.90
GLSN	Q03460	Glutamate synthase [NADH], amyloplastic	9.12
GSA	Q85WB7	Glutamate-1-semialdehyde 2,1-aminomutase, chloroplastic	3.58

GGAT2	Q9S7E9	Glutamate--glyoxylate aminotransferase 2	3.10
GLNA	P32289	Glutamine synthetase nodule isozyme	5.74
GLN2	O22506	Glutamine synthetase, chloroplastic	4.22
At3g24170	P48641	Glutathione reductase, cytosolic	3.23
GST	Q04522	Glutathione S-transferase	3.46
GSTP1	P09211	Glutathione S-transferase P	9.62
GSTZ	P57108	Glutathione S-transferase zeta class	-2.54
GDCSH	Q39732	Glycine cleavage system H protein, mitochondrial	3.61
HMT3	Q8LAX0	Homocysteine S-methyltransferase 3	-2.33
HGO	Q9ZRA2	Homogentisate 1,2-dioxygenase	-2.58
IGPS	P49572	Indole-3-glycerol phosphate synthase, chloroplastic	3.71
At3g58610	Q05758	Ketol-acid reductoisomerase, chloroplastic	3.72
LDHA	P00338	L-lactate dehydrogenase A chain	10.53
MGL	Q9SGU9	Methionine gamma-lyase	8.44
MTHFR2	O80585	Methylenetetrahydrofolate reductase 2	4.19
NNMT	P40261	Nicotinamide N-methyltransferase	9.12
ODC	O22616	Ornithine decarboxylase	4.23
MSRA	Q9SEC2	Peptide methionine sulfoxide reductase	4.04
MSRB2	Q9C5C8	Peptide methionine sulfoxide reductase B2, chloroplastic	9.39
SHKB	P37822	Phospho-2-dehydro-3-deoxyheptonate aldolase 2, chloroplastic	5.38
GSTX4	Q03666	Probable glutathione S-transferase	5.08
PAO4	Q8H191	Probable polyamine oxidase 4	4.91
POX1	P92983	Proline dehydrogenase 1, mitochondrial	6.07
WAT1	Q94AP3	Protein WALLS ARE THIN 1	5.29
Os02g0773300	Q6ZHE5	Putative D-cysteine desulfhydrase 1, mitochondrial	4.70
RIDA	Q94JQ4	Reactive Intermediate Deaminase A, chloroplastic	9.05
SAMDC	Q96555	S-adenosylmethionine decarboxylase proenzyme	9.59
SAMS	Q8GTL5	S-adenosylmethionine synthase	4.13
METK1	A9P822	S-adenosylmethionine synthase 1	4.17
SAMS2	Q96552	S-adenosylmethionine synthase 2	9.25
AGT1	Q56YA5	Serine--glyoxylate aminotransferase	3.90
TSB	O50046	Tryptophan synthase beta chain 2, chloroplastic	-1.08
**Lipid metabolism**			
PLSC	Q42670	1-acyl-sn-glycerol-3-phosphate acyltransferase	1.15
DWF5	Q9LDU6	7-dehydrocholesterol reductase	5.38
AKR1B1	P15121	Aldo-keto reductase family 1 member B1	4.06
ASPR	O04057	Aspartic proteinase	3.64
EPHX2	Q6Q2C2	Bifunctional epoxide hydrolase 2	1.09
APG	Q9LU14	GDSL esterase/lipase APG	3.77
At2g04570	Q9SJB4	GDSL esterase/lipase At2g04570	8.96
At4g01130	Q9M153	GDSL esterase/lipase At4g01130	3.61
At5g45670	Q9FK75	GDSL esterase/lipase At5g45670	3.93
INO1	Q9LW96	Inositol-3-phosphate synthase	1.86
PLC2	Q39033	Phosphoinositide phospholipase C 2	3.94
DALL3	Q3EBR6	Phospholipase A1-Igamma2, chloroplastic	-3.25
PLD1	Q41142	Phospholipase D alpha 1	3.68
pldY	Q54K50	Phospholipase D Y	3.29
CK1	Q9M9H6	Probable choline kinase 1	4.82
GPAT3	Q9SYJ2	Probable glycerol-3-phosphate acyltransferase 3	5.16
GPAT8	Q5XF03	Probable glycerol-3-phosphate acyltransferase 8	3.42
PDX1	Q39963	Probable pyridoxal 5'-phosphate synthase subunit PDX1	4.22
SQD2	Q8S4F6	Sulfoquinovosyl transferase SQD2	3.78
**Fatty acid metabolism**			
KCS1	Q9MAM3	3-ketoacyl-CoA synthase 1	2.98
CUT1	Q9XF43	3-ketoacyl-CoA synthase 6	4.54
FDH	Q570B4	3-ketoacyl-CoA synthase 10	4.21
KCS19	Q9LZ72	3-ketoacyl-CoA synthase 19	3.19
KCS20	Q9FG87	3-ketoacyl-CoA synthase 20	3.23
PED1	Q56WD9	3-ketoacyl-CoA thiolase 2, peroxisomal	5.19
fabG	Q9X248	3-oxoacyl-[acyl-carrier-protein] reductase FabG	1.51
ACP1	P93092	Acyl carrier protein 1, chloroplastic	-2.70
ACBP	Q39779	Acyl-CoA-binding protein	4.54
ACX4	Q96329	Acyl-coenzyme A oxidase 4, peroxisomal	9.47
AOS3	Q8GZP5	Allene oxide synthase 3	-3.70
MOD1	Q9SLA8	Enoyl-[acyl-carrier-protein] reductase [NADH], chloroplastic	3.52
HPL	K4CF70	Fatty acid hydroperoxide lyase, chloroplastic	6.42
FACT	Q9FFQ7	Fatty alcohol:caffeoyl-CoA acyltransferase	-2.33

LOX2.1	O24370	Linoleate 13S-lipoxygenase 2-1, chloroplastic	4.84
LOX3.1	O24371	Linoleate 13S-lipoxygenase 3-1, chloroplastic	3.34
LACS6	Q8LPS1	Long chain acyl-CoA synthetase 6, peroxisomal	5.16
LACS8	Q9SJD4	Long chain acyl-CoA synthetase 8	4.26
SMO1-2	Q1EC69	Methylsterol monooxygenase 1-2	8.67
FAD7	P48620	Omega-3 fatty acid desaturase, chloroplastic	4.74
FAD6C	P48627	Omega-6 fatty acid desaturase, chloroplastic	3.77
FATB1	Q9SQI3	Palmitoyl-acyl carrier protein thioesterase, chloroplastic	8.61
ADS3	Q949X0	Palmitoyl-monogalactosyldiacylglycerol delta-7 desaturase, chloroplastic	-2.33
ACX1	O65202	Peroxisomal acyl-coenzyme A oxidase 1	3.64
MFP2	Q9ZPI5	Peroxisomal fatty acid beta-oxidation multifunctional protein MFP2	3.38
Os02g0639600	Q6H5U3	Sphingolipid delta(4)-desaturase DES1-like	8.90
S-ACP-DES6	Q84VY3	Stearoyl-[acyl-carrier-protein] 9-desaturase 6, chloroplastic	-1.09
STAD	P22337	Stearoyl-[acyl-carrier-protein] 9-desaturase, chloroplastic	4.01
**Sulfur metabolism**			
AKN	O49204	Adenylyl-sulfate kinase, chloroplastic	3.69
APS1	Q9LIK9	ATP sulfurylase 1, chloroplastic	4.50
APS2	Q43870	ATP sulfurylase 2	4.35

Table S3. One hundred and fifty genes associated with other cell morphogenesis at NaCl (300 mmol/L) vs. CK.

Gene name	SwissProt ID	Protein name	log_2FC (NaCl vs. CK)
**Growth and development**			
AGRN	O00468	Agrin	8.86
AKR2A	Q9SAR5	Ankyrin repeat domain-containing protein 2A	3.55
AGP20	Q9M373	Arabinogalactan protein 20	3.35
CLPP4	Q94B60	ATP-dependent Clp protease proteolytic subunit 4, chloroplastic	4.54
CLPP6	Q9SAA2	ATP-dependent Clp protease proteolytic subunit 6, chloroplastic	4.84
CLPR1	Q9XJ35	ATP-dependent Clp protease proteolytic subunit-related protein 1	4.93
CNN3	Q15417	Calponin-3	8.54
MEE14	Q9XIM0	CCG-binding protein 1	3.37
CCN1	O00622	CCN family member 1	9.69
CCN2	P29279	CCN family member 2	4.75
CDC48	P54774	Cell division cycle protein 48 homolog	5.66
CPFTSY	O80842	Cell division protein FtsY homolog, chloroplastic	4.38
FTSZ2-1	O82533	Cell division protein FtsZ homolog 2-1, chloroplastic	4.80
CLPC	P35100	Chaperone protein ClpC, chloroplastic	3.86
CLPD	P42762	Chaperone protein ClpD, chloroplastic	5.36
ATJ8	Q9SAG8	Chaperone protein dnaJ 8, chloroplastic	4.25
ATJ11	Q9FYB5	Chaperone protein dnaJ 11, chloroplastic	4.07
COL1A1	P02452	Collagen alpha-1(I) chain	6.45
COL4A1	P02462	Collagen alpha-1(IV) chain	9.87
COL4A2	P08572	Collagen alpha-2(IV) chain	9.81
COL6A2	P12110	Collagen alpha-2(VI) chain	8.26
CYCD3-3	Q9SN11	Cyclin-D3-3	4.25
DUSP1	P28562	Dual specificity protein phosphatase 1	8.06
E134	Q9ZT66	Endo-1,3;1,4-beta-D-glucanase	-3.20
EBP1	M1CZC0	ERBB-3 BINDING PROTEIN 1	4.50
FLA1	Q9FM65	Fasciclin-like arabinogalactan protein 1	3.73
FLA7	Q9SJ81	Fasciclin-like arabinogalactan protein 7	9.41
FLA10	Q9LZX4	Fasciclin-like arabinogalactan protein 10	4.07
FLA11	Q8LEJ6	Fasciclin-like arabinogalactan protein 11	-1.19
FLA12	Q8LEE9	Fasciclin-like arabinogalactan protein 12	-1.05
FLA17	Q66GR0	Fasciclin-like arabinogalactan protein 17	4.14
FSTL1	Q12841	Follistatin-related protein 1	8.41
FRL4A	Q9LUV4	FRIGIDA-like protein 4a	4.09
SERPINE2	P07093	Glia-derived nexin	7.82
At5g07830	Q9FF10	Heparanase-like protein 1	8.74
IFRD1	Q5S1U6	Interferon-related developmental regulator 1	8.45
KRT19	P08727	Keratin, type I cytoskeletal 19	6.19
KRT8	P05787	Keratin, type II cytoskeletal 8	8.48
MMP14	P50281	Matrix metalloproteinase-14	4.54
VIPP1	O80796	Membrane-associated protein VIPP1, chloroplastic	9.87
2MMP	O04529	Metalloendoproteinase 2-MMP	-1.44
SKU5	Q9SU40	Monocopper oxidase-like protein SKU5	3.32

MYH9	P35579	Myosin-9	5.69
MYL12A	P19105	Myosin regulatory light chain 12A	4.87
AHNAK	Q09666	Neuroblast differentiation-associated protein AHNAK	4.98
SMG7	A9QM73	Nonsense-mediated mRNA decay factor SMG7	4.83
NPM1	P06748	Nucleophosmin	3.79
PATL3	Q56Z59	Patellin-3	3.03
At1g60420	O80763	Probable nucleoredoxin 1	10.27
Os01g0794400	Q0JIL1	Probable nucleoredoxin 2	4.24
CDI	Q9XIP8	Protein CDI	4.21
CHUP1	Q9LI74	Protein CHUP1, chloroplastic	3.79
CRWN1	F4HRT5	Protein CROWDED NUCLEI 1	3.40
CURT1A	O04616	Protein CURVATURE THYLAKOID 1A, chloroplastic	3.68
CURT1B	Q8LCA1	Protein CURVATURE THYLAKOID 1B, chloroplastic	3.52
HTH	Q9S746	Protein HOTHEAD	8.63
kra	Q9VNE2	Protein krasavietz	4.46
PHI-1	O82161	Protein PHOSPHATE-INDUCED 1	0.00
PTAC16	Q9STF2	Protein PLASTID TRANSCRIPTIONALLY ACTIVE 16, chloroplastic	3.88
REC2	F4JKH6	Protein REDUCED CHLOROPLAST COVERAGE 2	5.02
SEOB	Q9SS87	Protein SIEVE ELEMENT OCCLUSION B	8.35
SMXL7	O80875	Protein SMAX1-LIKE 7	4.43
At3g05500	Q9MA63	REF/SRPP-like protein At3g05500	9.29
RTN4	Q9NQC3	Reticulon-4	8.37
RTNLB2	Q9SUT9	Reticulon-like protein B2	5.93
SEPTIN2	Q15019	Septin-2	8.78
SPARC	P09486	SPARC	6.96
SIR1	O82802	Sulfite reductase 1 [ferredoxin], chloroplastic	3.36
TOM2A	Q9C5W7	Tobamovirus multiplication protein 2A	8.77
TAGLN	Q01995	Transgelin	9.16
TAGLN2	P37802	Transgelin-2	3.58
VCAN	P13611	Versican core protein	8.38
CER1	F4HVY0	Very-long-chain aldehyde decarbonylase CER1	-5.55
**Microtubule cytoskeleton organization**			
MAP65-1	Q9FLP0	65-kDa microtubule-associated protein 1	4.05
MAP65-6	Q9SIS3	65-kDa microtubule-associated protein 6	5.11
ACT	O81221	Actin	4.12
AC97	P30171	Actin-97	3.81
ADF1	Q9FVI2	Actin-depolymerizing factor 1	5.29
ADF2	Q9FVI1	Actin-depolymerizing factor 2	5.87
CAP1	Q01518	Adenylyl cyclase-associated protein 1	3.63
ACTN1	P12814	Alpha-actinin-1	5.29
ACTN4	O43707	Alpha-actinin-4	9.44
CFL1	P23528	Cofilin-1	3.71
CAP1	O65902	Cyclase-associated protein 1	8.73
FPP4	Q0WSY2	Filament-like plant protein 4	8.31
WLIM1	Q94JX5	LIM domain-containing protein WLIM1	9.33
MSN	P26038	Moesin	4.34
MYL6	P60661	Myosin light polypeptide 6	3.54
Pyy	Q9EPS2	Peptide YY	10.20
PRO1	O82572	Profilin-1	3.72
SP1L1	B3H4F1	Protein SPIRAL1-like 1	9.47
TMSB10	P63314	Thymosin beta-10	11.83
TPM4	P02561	Tropomyosin alpha-4 chain	9.89
TUBA	P33629	Tubulin alpha chain	6.31
TUBA1	P28752	Tubulin alpha-1 chain	3.60
TUBA1A	P68362	Tubulin alpha-1A chain	3.72
TUBA2	Q40832	Tubulin alpha-2 chain	-10.23
TUBA3	Q56WH1	Tubulin alpha-3 chain	3.69
TUBB1	P37392	Tubulin beta-1 chain	4.13
TUBB4B	Q3MHM5	Tubulin beta-4B chain	8.62
TUBB5	P46265	Tubulin beta-5 chain	3.47
VLN2	O81644	Villin-2	3.62
WDR1	O75083	WD repeat-containing protein 1	3.54
**Cell wall**			
ASD1	Q9SG80	Alpha-L-arabinofuranosidase 1	4.64
UGE1	Q42605	Bifunctional UDP-glucose 4-epimerase and UDP-xylose 4-epimerase 1	4.28
CALS3	Q9LXT9	Callose synthase 3	4.55
phiA	A2R2S8	Cell wall protein phiA	-9.90

CESA1	O48946	Cellulose synthase A catalytic subunit 1 [UDP-forming]	3.66
CESA2	O48947	Cellulose synthase A catalytic subunit 2 [UDP-forming]	3.84
CESA3	Q941L0	Cellulose synthase A catalytic subunit 3 [UDP-forming]	4.10
CSLG2	Q8VYR4	Cellulose synthase-like protein G2	2.88
EXPA1	Q9C554	Expansin-A1	4.90
EXPA4	O48818	Expansin-A4	3.77
EXPA8	O22874	Expansin-A8	-2.95
EXLA1	Q9LZT4	Expansin-like A1	4.02
GALS3	O65431	Galactan beta-1,4-galactosyltransferase GALS3	5.61
GDPDL3	Q9SZ11	Glycerophosphodiester phosphodiesterase GDPDL3	3.29
GDPDL4	Q9FJ62	Glycerophosphodiester phosphodiesterase GDPDL4	-1.16
LRX5	Q9SN46	Leucine-rich repeat extensin-like protein 5	3.06
MAN4	Q8L5J1	Mannan endo-1,4-beta-mannosidase 4	3.18
HHT1	Q94CD1	Omega-hydroxypalmitate O-feruloyl transferase	3.68
PAE8	Q6DBP4	Pectin acetylesterase 8	3.89
PECS-1.1	O04886	Pectinesterase 1	4.74
PECS-2.1	O04887	Pectinesterase 2	-1.39
MPE3	Q43111	Pectinesterase 3	-1.03
BXL2	Q94KD8	Probable beta-D-xylosidase 2	4.13
GATL1	Q9LN68	Probable galacturonosyltransferase-like 1	-1.05
GATL10	Q9LHD2	Probable galacturonosyltransferase-like 10	-1.76
GATL3	Q0V7R1	Probable galacturonosyltransferase-like 3	1.39
GATL7	Q8VYF4	Probable galacturonosyltransferase-like 7	5.23
XTH28	Q38909	Probable xyloglucan endotransglucosylase/hydrolase protein 28	3.57
XTH33	Q8LC45	Probable xyloglucan endotransglucosylase/hydrolase protein 33	-1.58
XTH6	Q8LF99	Probable xyloglucan endotransglucosylase/hydrolase protein 6	2.98
XTHB	Q8LNZ5	Probable xyloglucan endotransglucosylase/hydrolase protein B	3.61
IQD1	Q9SF32	Protein IQ-DOMAIN 1	4.40
IQD14	Q8LPG9	Protein IQ-DOMAIN 14	1.54
IPP	A2X8Q3	Soluble inorganic pyrophosphatase	6.10
PPA4	Q9LFF9	Soluble inorganic pyrophosphatase 4	4.54
PPA6	Q9LXC9	Soluble inorganic pyrophosphatase 6, chloroplastic	2.89
AXS2	Q9SGE0	UDP-D-apiose/UDP-D-xylose synthase 2	3.85
XTH22	Q38857	Xyloglucan endotransglucosylase/hydrolase protein 22	-1.22
XTH31	P93046	Xyloglucan endotransglucosylase/hydrolase protein 31	-3.75
XTH9	Q8LDW9	Xyloglucan endotransglucosylase/hydrolase protein 9	5.39
**Cell death**			
LGALS1	P09382	Galectin-1	10.68
GAS6	Q14393	Growth arrest-specific protein 6	5.53
MT-RNR2	Q8IVG9	Humanin	8.21
IGFBP3	P17936	Insulin-like growth factor-binding protein 3	8.58
IGFBP4	P22692	Insulin-like growth factor-binding protein 4	6.91
IGFBP7	Q16270	Insulin-like growth factor-binding protein 7	10.58
MAGED1	Q9Y5V3	Melanoma-associated antigen D1	8.21

Table S4. Other forty six TFs at NaCl (300 mmol/L) vs. CK.

Gene name	SwissProt ID	Protein name	log_2FC (NaCl vs. CK)
COL4	Q940T9	Zinc finger protein CONSTANS-LIKE 4	2.76
COL9	Q9SSE5	Zinc finger protein CONSTANS-LIKE 9	3.40
COL15	Q9C7E8	Zinc finger protein CONSTANS-LIKE 15	3.63
COL16	Q8RWD0	Zinc finger protein CONSTANS-LIKE 16	3.98
Os01g0871200	Q943I6	Zinc finger protein STOP1 homolog	8.92
ZFP6	Q39265	Zinc finger protein 6	-4.21
ZAT6	O22533	Zinc finger protein ZAT6	-1.51
ZAT10	Q96289	Zinc finger protein ZAT10	5.60
MIF2	Q9LJW5	Mini zinc finger protein 2	-1.01
MIP1B	Q9LRM4	B-box domain protein 31	1.94
BBX19	C0SVM5	B-box zinc finger protein 19	1.25
BLH1	Q9SJ56	BEL1-like homeodomain protein 1	4.15
BLH7	Q9SIW1	BEL1-like homeodomain protein 7	9.02
CIP7	O80386	COP1-interacting protein 7	3.28
DCN	P07585	Decorin	8.51
GATA8	Q9SV30	GATA transcription factor 8	3.18
GBF1	P42774	G-box-binding factor 1	4.49
HMGA1	Q6URC2	High mobility group protein HMG-	5.81

		I/HMG-Y	
KNAP3	O04136	Homeobox protein knotted-1-like 3	3.78
ATHB-7	P46897	Homeobox-leucine zipper protein ATHB-7	2.23
ATHB-12	Q9M276	Homeobox-leucine zipper protein ATHB-12	1.23
ATHB-16	Q940J1	Homeobox-leucine zipper protein ATHB-16	3.40
HAT22	P46604	Homeobox-leucine zipper protein HAT22	1.36
HOX16	A2X980	Homeobox-leucine zipper protein HOX16	4.45
HIF1A	Q16665	Hypoxia-inducible factor 1-alpha	9.20
LBD4	Q9SHE9	LOB domain-containing protein 4	1.20
LBD15	Q8L5T5	LOB domain-containing protein 15	-3.06
LBD18	O22131	LOB domain-containing protein 18	-2.08
LOXL2	Q9Y4K0	Lysyl oxidase homolog 2	9.91
MBF1A	Q9SJI8	Multiprotein-bridging factor 1a	3.33
AFP3	Q94F39	Ninja-family protein AFP3	1.33
NFYA4	Q8VY64	Nuclear transcription factor Y subunit A-4	1.93
PAX8	Q06710	Paired box protein Pax-8	8.42
CAF1-9	Q9LXM2	Probable CCR4-associated factor 1 homolog 9	9.59
CAF1-11	Q9FMS6	Probable CCR4-associated factor 1 homolog 11	-1.50
GLK1	Q5Z5I4	Probable transcription factor GLK1	3.82
LHY	Q6R0H1	Protein LHY	1.07
PTMA	P06454	Prothymosin alpha	8.86
SCL13	Q9M0M5	Scarecrow-like protein 13	3.82
TPR1	Q0WV90	Topless-related protein 1	3.99
Os07g0631100	Q8LHP0	Transcription elongation factor 1 homolog	10.12
CSA	Q5NBM8	Transcription factor CSA	3.36
DIVARICATA	Q8S9H7	Transcription factor DIVARICATA	9.63
GTE7	Q7Y214	Transcription factor GTE7	4.35
MYC2	A0A3Q7HRZ6	Transcription factor MYC2	3.81
GT-2	Q39117	Trihelix transcription factor GT-2	4.70

Table S5. One hundred and thirty-seven genes associated with other bio-signaling at NaCl (300 mmol/L) vs.CK.

**Gene name**	**SwissProt ID**	**Protein name**	**log_2FC (NaCl vs. CK)**
**Calcium ion**			
RCAN2	Q14206	Calcipressin-2	-9.14
CBP	Q2QY10	Calcium-binding protein CBP	9.34
KRP1	O81831	Calcium-binding protein KRP1	-1.63
cam	A8CEP3	Calmodulin	-10.18
CALM1	P0DP23	Calmodulin-1	5.14
CAM-1	P62200	Calmodulin-1/11/16	4.80
CBP60A	C0SVV6	Calmodulin-binding protein 60 A	3.28
CBP60B	Q9FKL6	Calmodulin-binding protein 60 B	2.98
CANX	P27824	Calnexin	8.62
CALX	Q39817	Calnexin homolog	3.24
CAPN2	P17655	Calpain-2 catalytic subunit	5.17
CALU	O43852	Calumenin	8.69
CML13	Q94AZ4	Probable calcium-binding protein CML13	5.09
CML16	Q9LI84	Probable calcium-binding protein CML16	1.30
CML18	Q0DJV6	Probable calcium-binding protein CML18	-1.13
CML27	Q9LE22	Probable calcium-binding protein CML27	10.02
S100A6	P06703	Protein S100-A6	3.33
S100A16	Q96FQ6	Protein S100-A16	8.91
**Protein kinase**			
CPK6	Q38872	Calcium-dependent protein kinase 6	3.97
CPK19	P53683	Calcium-dependent protein kinase 19	9.29
CPK28	Q9FKW4	Calcium-dependent protein kinase 28	4.19
CPK32	Q6NLQ6	Calcium-dependent protein kinase 32	3.23
CIPK6	O65554	CBL-interacting serine/threonine-protein kinase 6	4.52
CIPK25	Q8W1D5	CBL-interacting serine/threonine-protein kinase 25	4.86
CDKF-4	Q6Z8C8	Cyclin-dependent kinase F-4	3.87
CDKG-2	A2XUW1	Cyclin-dependent kinase G-2	6.65

CRK8	O65468	Cysteine-rich receptor-like protein kinase 8	5.39
CRK26	Q9T0J1	Cysteine-rich receptor-like protein kinase 26	4.87
MPK3	Q39023	Mitogen-activated protein kinase 3	-1.67
MPK17	Q84M93	Mitogen-activated protein kinase 17	9.31
MAPKKK3	F4HRJ4	Mitogen-activated protein kinase kinase kinase 3	8.64
At5g24010	Q9FLW0	Probable receptor-like protein kinase At5g24010	3.24
At1g01540	Q3EDL4	Probable serine/threonine-protein kinase At1g01540	3.10
At1g54610	Q9ZVM9	Probable serine/threonine-protein kinase At1g54610	8.29
SIS8	Q9C9U5	Probable serine/threonine-protein kinase SIS8	3.54
At5g49770	Q9LT96	Probable leucine-rich repeat receptor-like protein kinase At5g49770	3.91
PTI11	Q8H1G6	PTI1-like tyrosine-protein kinase 1	3.32
RLK7	F4I2N7	Receptor-like protein kinase 7	8.52
FER	Q9SCZ4	Receptor-like protein kinase FERONIA	6.05
THE1	Q9LK35	Receptor-like protein kinase THESEUS 1	1.10
BSK1	Q944A7	Serine/threonine-protein kinase BSK1	3.60
CTR1	Q05609	Serine/threonine-protein kinase CTR1	-2.32
MHK	P43294	Serine/threonine-protein kinase MHK	8.59
PBL35	Q9SRH7	Serine/threonine-protein kinase PBL35	8.52
PLK2	Q9NYY3	Serine/threonine-protein kinase PLK2	8.72
SAPK10	Q75H77	Serine/threonine-protein kinase SAPK10	8.37
SRK2A	P43291	Serine/threonine-protein kinase SRK2A	4.38
STY13	Q9ZQ31	Serine/threonine-protein kinase STY13	3.30
STY46	F4JTP5	Serine/threonine-protein kinase STY46	3.30
CCR4	Q9FIJ6	Serine/threonine-protein kinase-like protein CCR4	-1.43
ASK1	P43288	Shaggy-related protein kinase alpha	3.81
ASK10	Q39019	Shaggy-related protein kinase kappa	4.58
KIN10	Q38997	SNF1-related protein kinase catalytic subunit alpha KIN10	4.11
KING1	Q8LBB2	SNF1-related protein kinase regulatory subunit gamma-1	3.29
**Protein phosphatase**			
At2g29380	Q9ZW21	Probable protein phosphatase 2C 24	1.50
Os04g0609600	Q0JAA0	Probable protein phosphatase 2C 44	8.69
Os06g0717800	Q5Z8P0	Probable protein phosphatase 2C 60	3.90
At5g53140	Q94AT1	Probable protein phosphatase 2C 76	5.08
SRF6	Q9C8M9	Protein STRUBBELIG-RECEPTOR FAMILY 6	8.68
PAP2	Q9SDZ9	Purple acid phosphatase 2	5.06
PAP18	Q9LJU7	Purple acid phosphatase 18	4.89
PPP2R1A	Q32PI5	Serine/threonine-protein phosphatase 2A 65 kDa regulatory subunit A alpha isoform	4.91
PP2AA2	Q38950	Serine/threonine-protein phosphatase 2A 65 kDa regulatory subunit A beta isoform	3.24
PPP6R3	Q5F471	Serine/threonine-protein phosphatase 6 regulatory subunit 3	3.78
**Others**			
TFT7	P93212	14-3-3 protein 7	3.77
YWHAE	P62261	14-3-3 protein epsilon	9.13
YWHAQ	Q3SZI4	14-3-3 protein theta	4.32
YWHAZ	P63103	14-3-3 protein zeta/delta	4.06
GF14	P46266	14-3-3-like protein	3.63
GF14A	Q96450	14-3-3-like protein A	2.60
GF14D	Q96453	14-3-3-like protein D	4.01
21KD	P17407	21 kDa protein	-1.32
ASP	P32765	21 kDa seed protein	4.06
CPN20	O65282	20 kDa chaperonin, chloroplastic	3.19
Alb	P07724	Albumin	-6.57
Serpina1c	Q00896	Alpha-1-antitrypsin 1-3	-12.26
APLP2	Q06481	Amyloid beta precursor like protein 2	5.13
APP	P05067	Amyloid-beta precursor protein	9.34
ANXA2	P07355	Annexin A2	7.20
ANXA5	P08758	Annexin A5	4.01
pstI	P08594	Aqualysin-1	-10.86
CKL1	P42158	Casein kinase 1-like protein 1	4.17
CKL2	Q9CAI5	Casein kinase 1-like protein 2	4.21
HD16	Q852L0	Casein kinase 1-like protein HD16	4.33
CBBY	Q94K71	CBBY-like protein	4.59
CD81	P60033	CD81 antigen	5.05
CD59	P13987	CD59 glycoprotein	5.12
CD151	P48509	CD151 antigen	9.74
DKK3	Q9UBP4	Dickkopf-related protein 3	8.44

DCBLD2	Q96PD2	Discoidin, CUB and LCCL domain-containing protein 2	9.74
ERG3	Q25AG5	Elicitor-responsive protein 3	-1.37
FN1	P02751	Fibronectin	11.00
FHL2	Q14192	Four and a half LIM domains protein 2	5.15
LGALS3BP	Q08380	Galectin-3-binding protein	5.45
Gnat1	P20612	Guanine nucleotide-binding protein G(t) subunit alpha-1	-11.53
GNB1	P62871	Guanine nucleotide-binding protein G(I)/G(S)/G(T) subunit beta-1	0.89
GNB2L1	Q4R7Y4	Guanine nucleotide-binding protein subunit beta-2-like 1	4.86
GNBL	Q39836	Guanine nucleotide-binding protein subunit beta-like protein	4.16
NSN1	Q9M8Z5	Guanine nucleotide-binding protein-like NSN1	8.68
GDI1	Q96254	Guanosine nucleotide diphosphate dissociation inhibitor 1	4.38
ITM2B	Q9Y287	Integral membrane protein 2B	9.73
ITGA3	P26006	Integrin alpha-3	9.81
ITGB1	P05556	Integrin beta-1	7.79
Iapp	P12968	Islet amyloid polypeptide	13.78
pKIWI504	P43390	Metallothionein-like protein type 2	3.58
MIP1	Q8RX22	MND1-interacting protein 1	-2.95
NRG2	Q93YU8	Nitrate regulatory gene2 protein	9.06
Ppy	P10601	Pancreatic prohormone	11.60
Prph2	P15499	Peripherin-2	-7.68
PI4KG7	Q9SI52	Phosphatidylinositol 4-kinase gamma 7	2.96
PHOT1	O48963	Phototropin-1	2.87
PHOT2	P93025	Phototropin-2	3.84
PMP3	Q4HXT6	Plasma membrane proteolipid 3	-9.84
PCAP1	Q96262	Plasma membrane-associated cation-binding protein 1	4.31
SERPINE1	P05121	Plasminogen activator inhibitor 1	11.40
AGD8	Q8H100	Probable ADP-ribosylation factor GTPase-activating protein AGD8	3.93
PAP27	Q5MAU8	Probable inactive purple acid phosphatase 27	5.36
PDIA6	P38661	Probable protein disulfide-isomerase A6	3.75
At3g55350	Q9M2U3	Protein ALP1-like	3.76
TIC	Q94KE2	Protein TIME FOR COFFEE	4.52
At5g47540	Q9FGK3	Putative MO25-like protein At5g47540	3.26
REN1	F4JQZ3	Rho GTPase-activating protein REN1	-1.19
Rho	P15409	Rhodopsin	-9.76
Rom1	P32958	Rod outer segment membrane protein 1	-9.73
Sag	P20443	S-arrestin	-9.73
SPP	O81062	Signal peptide peptidase	5.08
SPPL2	Q8W469	Signal peptide peptidase-like 2	4.36
D14	Q9SQR3	Strigolactone esterase D14	5.00
BRI1	Q8L899	Systemin receptor SR160	5.12
THBS1	P07996	Thrombospondin-1	8.22
THY1	P04216	Thy-1 membrane glycoprotein	9.18
TGFBI	Q15582	Transforming growth factor-beta-induced protein ig-h3	5.50
RHOA	P61585	Transforming protein RhoA	3.89
TPT1	P13693	Translationally-controlled tumor protein	5.79
TCTP	Q5J907	Translationally-controlled tumor protein homolog	4.68
APRR1	Q9LKL2	Two-component response regulator-like APRR1	3.64
PLAU	P00749	Urokinase-type plasminogen activator	9.82

Table S6. One hundred and ten genes associated with transport at NaCl (300 mmol/L) vs. CK.

Gene name	SwissProt ID	Protein name	log_2FC (NaCl vs. CK)
**Sugar**			
At5g17010	Q6AWX0	D-xylose-proton symporter-like 2	5.29
At5g59250	Q0WWW9	D-xylose-proton symporter-like 3, chloroplastic	8.93
At5g16150	Q56ZZ7	Plastidic glucose transporter 4	5.80
SUC2	Q39231	Sucrose transport protein SUC2	3.12
STP13	Q94AZ2	Sugar transport protein 13	9.64
At5g18840	Q8LBI9	Sugar transporter ERD6-like 16	-3.19
**Protein**			
aac	P02723	ADP,ATP carrier protein	-9.69
ANT1	O22342	ADP,ATP carrier protein 1, mitochondrial	3.60
AAC3	O49447	ADP,ATP carrier protein 3, mitochondrial	4.94
ARF	O48920	ADP-ribosylation factor	4.31
arf1	P36579	ADP-ribosylation factor 1	-10.21
ARF2	P51823	ADP-ribosylation factor 2	3.85

AGD5	Q9FL69	ADP-ribosylation factor GTPase-activating protein AGD5	4.69
SNAA	P93798	Alpha-soluble NSF attachment protein	4.72
AP2M1	Q3ZC13	AP-2 complex subunit mu	5.08
RAN3	Q8H156	GTP-binding nuclear protein Ran-3	5.12
EMB2001	O81004	GTP-binding protein At2g22870	8.62
SAR1B	Q01474	GTP-binding protein SAR1B	4.38
YPTM2	Q05737	GTP-binding protein YPTM2	4.21
IMPA1	Q96321	Importin subunit alpha-1	3.55
NACA	Q13765	Nascent polypeptide-associated complex subunit alpha	9.42
NACA1	Q9M612	Nascent polypeptide-associated complex subunit alpha-like protein	-10.00
At1g73230	Q9CAT7	Nascent polypeptide-associated complex subunit beta	3.39
OPT3	O23482	Oligopeptide transporter 3	1.03
SFH8	F4IHJ0	Phosphatidylinositol/phosphatidylcholine transfer protein SFH8	6.71
TIC55	O49931	Protein TIC 55, chloroplastic	4.92
TIC62	Q8H0U5	Protein TIC 62, chloroplastic	3.20
TIC110	Q8LPR9	Protein TIC110, chloroplastic	4.52
SecA	Q41062	Protein translocase subunit SecA, chloroplastic	2.71
sec61a	Q54XK2	Protein transport protein Sec61 subunit alpha	4.01
SCAMP1	A2YMP7	Putative secretory carrier-associated membrane protein 1	3.56
RAB7	P31022	Ras-related protein Rab7	9.54
RABC2A	O49841	Ras-related protein RABC2a	1.38
RABE1C	P28186	Ras-related protein RABE1c	3.68
RABH1B	O80501	Ras-related protein RABH1b	5.33
SYP121	Q9ZSD4	Syntaxin-121	-1.18
SYP51	Q9SA23	Syntaxin-51	5.58
TOC120	Q9LUS2	Translocase of chloroplast 120, chloroplastic	8.63
VAMP725	O48850	Vesicle-associated membrane protein 725	3.83
**Amino acid**			
AVT3C	Q9SVG0	Amino acid transporter AVT3C	3.18
AVT6A	Q9LI61	Amino acid transporter AVT6A	3.44
BAT1	Q9ZU50	Amino-acid permease BAT1	2.96
CAT2	Q9ASS7	Cationic amino acid transporter 2, vacuolar	4.24
CAT7	Q9SQZ0	Cationic amino acid transporter 7, chloroplastic	4.68
LHT1	Q9FKS8	Lysine histidine transporter 1	9.46
YPQ1	Q12010	Probable vacuolar amino acid transporter YPQ1	-1.98
**lipid**			
MALD3	Q9M5X7	Non-specific lipid-transfer protein	3.69
LTP1	O24037	Non-specific lipid-transfer protein 1	1.07
LTPG1	Q9C7F7	Non-specific lipid transfer protein GPI-anchored 1	10.13
LSAT_1X82001	A0A2J6KL39	Non-specific lipid-transfer protein Lac s 1	3.50
SYT1	Q9SKR2	Synaptotagmin-1	3.52
SYT4	A0JJX5	Synaptotagmin-4	5.85
**ABC**			
ABCB4	O80725	ABC transporter B family member 4	9.11
ABCB11	Q9FWX7	ABC transporter B family member 11	-1.42
ABCC1	Q9C8G9	ABC transporter C family member 1	4.33
ABCC2	Q42093	ABC transporter C family member 2	5.06
ABCC5	Q7GB25	ABC transporter C family member 5	-2.29
ABCF1	Q9FJH6	ABC transporter F family member 1	4.65
ABCF5	Q9LV93	ABC transporter F family member 5	3.36
ABCG7	Q9ZU35	ABC transporter G family member 7	2.87
ABCG20	Q9LFG8	ABC transporter G family member 20	2.36
ABCG36	Q9XIE2	ABC transporter G family member 36	3.40
**Others**			
Apoa1	Q00623	Apolipoprotein A-I	-7.20
PIP1-5	Q9AR14	Aquaporin PIP1-5	3.66
TIP1-1	P25818	Aquaporin TIP1-1	1.27
TIP2-1	Q41951	Aquaporin TIP2-1	4.40
SWEET16	Q10LN5	Bidirectional sugar transporter SWEET16	1.50
CHC1	Q0WNJ6	Clathrin heavy chain 1	4.00
At1g62020	Q94A40	Coatomer subunit alpha-1	3.56
At1g52360	Q9C827	Coatomer subunit beta'-2	3.00
DIT1	Q9LXV3	Dicarboxylate transporter 1, chloroplastic	5.01
EP1	Q39688	Epidermis-specific secreted glycoprotein EP1	-0.88
CHMP1A	Q8LE58	ESCRT-related protein CHMP1A	5.55
EXO70A1	Q9LZD3	Exocyst complex component EXO70A1	-1.65
EXO70A3	F4KG58	Exocyst complex component EXO70A3	-1.97

KHSRP	Q8UVD9	Far upstream element-binding protein 2	3.23
FUBP3	Q96I24	Far upstream element-binding protein 3	3.25
HIPP26	Q9SZN7	Heavy metal-associated isoprenylated plant protein 26	2.95
Hgsnat	Q3UDW8	Heparan-alpha-glucosaminide N-acetyltransferase	4.21
Ipo5	Q8BKC5	Importin-5	8.97
INT1	Q8VZR6	Inositol transporter 1	1.19
NAT6	Q27GI3	Nucleobase-ascorbate transporter 6	3.84
ATAD1	F6QV99	Outer mitochondrial transmembrane helix translocase	3.96
AZI1	Q9SU35	pEARLI1-like lipid transfer protein 1	3.67
PMA4	Q03194	Plasma membrane ATPase 4	3.45
PLGG1	Q9FVQ4	Plastidal glycolate/glycerate translocator 1, chloroplastic	5.77
TLC1	O24381	Plastidic ATP/ADP-transporter	3.20
SCY1	Q38885	Preprotein translocase subunit SCY1, chloroplastic	4.11
PIP1-2	Q7XSQ9	Probable aquaporin PIP1-2	3.92
PIP2-5	Q9SV31	Probable aquaporin PIP2-5	3.81
INT2	Q9C757	Probable inositol transporter 2	1.23
SULTR4;2	Q8GYH8	Probable sulfate transporter 4.2	3.87
DTX51	Q9SZE2	Protein DETOXIFICATION 51	-2.31
DTX54	Q9LE20	Protein DETOXIFICATION 54	1.32
NPF1.2	Q9M817	Protein NRT1/ PTR FAMILY 1.2	3.71
NPF2.10	Q944G5	Protein NRT1/ PTR FAMILY 2.10	1.32
NPF6.4	Q9LVE0	Protein NRT1/ PTR FAMILY 6.4	4.75
NPF7.3	Q9LQL2	Protein NRT1/ PTR FAMILY 7.3	1.19
NPF8.3	P46032	Protein NRT1/ PTR FAMILY 8.3	4.27
SKD1	Q9ZNT0	Protein SUPPRESSOR OF K(+) TRANSPORT GROWTH DEFECT 1	4.26
TIC100	Q8LPR8	Protein TIC 100	3.83
ALY1	Q8L773	THO complex subunit 4A	5.83
TMN2	Q940S0	Transmembrane 9 superfamily member 2	9.34
TMN4	Q9FHT4	Transmembrane 9 superfamily member 4	3.77
TMN7	Q9LIC2	Transmembrane 9 superfamily member 7	4.59
TMN8	F4KIB2	Transmembrane 9 superfamily member 8	3.49
TMN12	F4JRE0	Transmembrane 9 superfamily member 12	4.32
TMED9	Q9BVK6	Transmembrane emp24 domain-containing protein 9	8.59
tmem184C	Q54WM0	Transmembrane protein 184 homolog DDB_G0279555	8.41
TPT	Q9ZSR7	Triose phosphate/phosphate translocator TPT, chloroplastic	3.97

Table S7. One hundred and twenty-five genes associated with photosynthesis and energy at NaCl (300 mmol/L) vs. CK.

Gene name	SwissProt ID	Protein name	$\log_2$FC (NaCl vs. CK)
**Photosynthesis**			
PHR2	Q8LB72	Blue-light photoreceptor PHR2	3.13
CAB12	P13869	Chlorophyll a-b binding protein, chloroplastic	4.08
CAB13	P27489	Chlorophyll a-b binding protein 13, chloroplastic	3.39
CAB21	P27493	Chlorophyll a-b binding protein 21, chloroplastic	3.72
CAB-151	P27518	Chlorophyll a-b binding protein 151, chloroplastic	3.16
CAP10A	P27524	Chlorophyll a-b binding protein CP24 10A, chloroplastic	2.84
LHCA1	Q01667	Chlorophyll a-b binding protein 6, chloroplastic	3.75
LHCB5	Q9XF89	Chlorophyll a-b binding protein CP26, chloroplastic	4.12
LHCB4.1	Q07473	Chlorophyll a-b binding protein CP29.1, chloroplastic	4.36
LHCB4.3	Q9S7W1	Chlorophyll a-b binding protein CP29.3, chloroplastic	4.14
lhcA-P4	Q9SQL2	Chlorophyll a-b binding protein P4, chloroplastic	4.12
ELI	P11432	Early light-induced protein, chloroplastic	1.11
CHLP	Q9ZS34	Geranylgeranyl diphosphate reductase, chloroplastic	4.11
GGPS	Q94ID7	Geranylgeranyl pyrophosphate synthase, chloroplastic	3.45
HEMA1	P93111	Glutamyl-tRNA reductase 1, chloroplastic	5.00
HPR-A	P13443	Glycerate dehydrogenase	3.88
GOX	P05414	Glycolate oxidase	3.77
LIL3.1	Q9SYX1	Light-harvesting complex-like protein 3 isotype 1, chloroplastic	3.93
LIPC	P80471	Light-induced protein, chloroplastic	3.94
LIR1	Q96500	Light-regulated protein 1, chloroplastic	3.72
CRTL-E-1	O65837	Lycopene epsilon cyclase, chloroplastic	3.36
CHLM	Q9SW18	Magnesium protoporphyrin IX methyltransferase, chloroplastic	4.39
CHLD	O24133	Magnesium-chelatase subunit ChlD, chloroplastic	3.55
CHLH	Q9FNB0	Magnesium-chelatase subunit ChlH, chloroplastic	3.46

CHLI	P93162	Magnesium-chelatase subunit ChlI, chloroplastic	2.70
CRD1	Q6SJV8	Magnesium-protoporphyrin IX monomethyl ester [oxidative] cyclase	3.78
PSBO	Q40459	Oxygen-evolving enhancer protein 1, chloroplastic	3.89
PSBP	P93566	Oxygen-evolving enhancer protein 2, chloroplastic	4.07
PSBQ	P12301	Oxygen-evolving enhancer protein 3, chloroplastic	3.33
PGRL1A	Q8H112	PGR5-like protein 1A, chloroplastic	3.85
PGLP1A	P0DKC3	Phosphoglycolate phosphatase 1A, chloroplastic	3.85
LHCA3	Q9SY97	Photosystem I chlorophyll a/b-binding protein 3-1, chloroplastic	3.51
LHCA5	Q9C639	Photosystem I chlorophyll a/b-binding protein 5, chloroplastic	3.18
LHCA6	Q8LCQ4	Photosystem I chlorophyll a/b-binding protein 6, chloroplastic	3.99
psaD	P32869	Photosystem I reaction center subunit II, chloroplastic	3.95
PSAF	Q9SHE8	Photosystem I reaction center subunit III, chloroplastic	3.55
PSAEA	Q41228	Photosystem I reaction center subunit IV A, chloroplastic	3.25
PSAN	P49107	Photosystem I reaction center subunit N, chloroplastic	4.54
PSAK	Q9SUI5	Photosystem I reaction center subunit psaK, chloroplastic	3.80
PSAG	Q9S7N7	Photosystem I reaction center subunit V, chloroplastic	3.22
PSAH2	Q9SUI6	Photosystem I reaction center subunit VI-2, chloroplastic	3.86
PSAL	Q9SUI4	Photosystem I reaction center subunit XI, chloroplastic	4.02
PSAO	Q949Q5	Photosystem I subunit O	3.64
PSBR	P10690	Photosystem II 10 kDa polypeptide, chloroplastic	4.11
PSBS	Q02060	Photosystem II 22 kDa protein, chloroplastic	3.43
PSBT	P31336	Photosystem II 5 kDa protein, chloroplastic	3.22
PSBY	P80470	Photosystem II core complex proteins psbY, chloroplastic	3.90
PSBW	Q39194	Photosystem II reaction center W protein, chloroplastic	3.50
PSB27-1	Q9LR64	Photosystem II repair protein PSB27-H1, chloroplastic	4.00
PSY	P49293	Phytoene synthase, chloroplastic	4.29
CCD4	O49675	Probable carotenoid cleavage dioxygenase 4, chloroplastic	2.93
NYC1	Q5N800	Probable chlorophyll(ide) b reductase NYC1, chloroplastic	4.49
ABC1K1	Q8RWG1	Protein ACTIVITY OF BC1 COMPLEX KINASE 1, chloroplastic	4.27
ABC1K3	Q9MA15	Protein ACTIVITY OF BC1 COMPLEX KINASE 3, chloroplastic	3.41
ABC1K8	Q93Y08	Protein ACTIVITY OF BC1 COMPLEX KINASE 8, chloroplastic	4.08
CYP97A3	Q93VK5	Protein LUTEIN DEFICIENT 5, chloroplastic	5.90
SGR	A9YF60	Protein STAY-GREEN homolog, chloroplastic	-2.32
THF1	Q7XAB8	Protein THYLAKOID FORMATION1, chloroplastic	4.26
PORA	Q41249	Protochlorophyllide reductase, chloroplastic	3.76
PPD3	Q9S720	PsbP domain-containing protein 3, chloroplastic	9.12
PYG7	B9DHG0	Tetratricopeptide repeat domain-containing protein PYG7, chloroplastic	8.75
At1g54780	Q9ZVL6	UPF0603 protein At1g54780, chloroplastic	4.27
VDE1	Q9SM43	Violaxanthin de-epoxidase, chloroplastic	3.89
ZDS	Q9FV46	Zeta-carotene desaturase, chloroplastic/chromoplastic	4.15
**Energy**			
DBR	Q9SLN8	2-alkenal reductase (NADP(+)-dependent)	8.73
adh-1	Q9P6C8	Alcohol dehydrogenase 1	-9.86
FTSH	Q9BAE0	ATP-dependent zinc metalloprotease FTSH, chloroplastic	3.26
FTSH2	O80860	ATP-dependent zinc metalloprotease FTSH 2, chloroplastic	4.22
FTSH6	Q1PDW5	ATP-dependent zinc metalloprotease FTSH 6, chloroplastic	1.39
FTSH11	Q9FGM0	ATP-dependent zinc metalloprotease FTSH 11, chloroplastic/mitochondrial	3.81
ATPD	P32980	ATP synthase delta chain, chloroplastic	3.65
ATP5F1A	P25705	ATP synthase subunit alpha, mitochondrial	4.32
ATPF2	P31853	ATP synthase subunit b', chloroplastic	4.62
ATPB	P29685	ATP synthase subunit beta, mitochondrial	3.88
At3g52300	Q9FT52	ATP synthase subunit d, mitochondrial	4.11
ATP4	Q40089	ATP synthase subunit delta', mitochondrial	4.58
Atp5f1e	P56382	ATP synthase subunit epsilon, mitochondrial	-10.09
Atp5mf	P56135	ATP synthase subunit f, mitochondrial	-3.56
ATPC	P26360	ATP synthase subunit gamma, mitochondrial	4.32
CEQORH	Q9SV68	Chloroplast envelope quinone oxidoreductase homolog	9.51
MT-CYB	P00156	Cytochrome b	12.19
CYTB5-E	Q42342	Cytochrome b5 isoform E	9.19
petC	P08980	Cytochrome b6-f complex iron-sulfur subunit, chloroplastic	3.77
petM	P80883	Cytochrome b6-f complex subunit 7	3.36
QCR7-2	F4JWS8	Cytochrome b-c1 complex subunit 7-2, mitochondrial	4.82
MT-CO1	P00395	Cytochrome c oxidase subunit 1	7.30
MT-CO2	P00403	Cytochrome c oxidase subunit 2	13.22
MT-CO3	P00414	Cytochrome c oxidase subunit 3	8.37
COX5B-1	Q9LW15	Cytochrome c oxidase subunit 5b-1, mitochondrial	5.57

At4g27520	Q9T076	Early nodulin-like protein 2	4.35
FDC2	Q9C7Y4	Ferredoxin C 2, chloroplastic	9.52
FD1	O04090	Ferredoxin-1, chloroplastic	3.93
GLU1	Q9ZNZ7	Ferredoxin-dependent glutamate synthase 1, chloroplastic/mitochondrial	3.74
LFNR1	Q9FKW6	Ferredoxin--NADP reductase, leaf isozyme 1, chloroplastic	4.09
FENR2	O04397	Ferredoxin--NADP reductase, root-type isozyme, chloroplastic	4.10
NIR1	P38500	Ferredoxin--nitrite reductase, chloroplastic	3.48
FTRV	P80680	Ferredoxin-thioredoxin reductase, variable chain	3.14
FRO7	Q3KTM0	Ferric reduction oxidase 7, chloroplastic	2.38
FRI3	Q948P6	Ferritin-3, chloroplastic	4.29
ADNT1	O04619	Mitochondrial adenine nucleotide transporter ADNT1	4.94
VDAC2	P42056	Mitochondrial outer membrane protein porin of 36 kDa	4.18
PUMP5	Q9SJY5	Mitochondrial uncoupling protein 5	2.84
MPP	P29677	Mitochondrial-processing peptidase subunit alpha	3.70
ndhM	A9PJQ8	NAD(P)H-quinone oxidoreductase subunit M, chloroplastic	4.40
At3g08610	Q9C9Z5	NADH dehydrogenase [ubiquinone] 1 alpha subcomplex subunit 1	5.14
At2g20360	Q9SK66	NADH dehydrogenase [ubiquinone] 1 alpha subcomplex subunit 9	5.80
At5g08530	Q9FNN5	NADH dehydrogenase [ubiquinone] flavoprotein 1, mitochondrial	3.68
N/A	Q43644	NADH dehydrogenase [ubiquinone] iron-sulfur protein 1, mitochondrial	4.23
MT-ND1	P03886	NADH-ubiquinone oxidoreductase chain 1	12.21
MT-ND2	P03891	NADH-ubiquinone oxidoreductase chain 2	7.93
MT-ND4	P03905	NADH-ubiquinone oxidoreductase chain 4	9.39
MT-ND5	P03915	NADH-ubiquinone oxidoreductase chain 5	9.11
GAPN	P93338	NADP-dependent glyceraldehyde-3-phosphate dehydrogenase	4.57
NADP-ME4	Q9CA83	NADP-dependent malic enzyme 4, chloroplastic	3.46
PNSL5	Q9ASS6	Photosynthetic NDH subunit of lumenal location 5, chloroplastic	4.39
PNSB1	Q9S9N6	Photosynthetic NDH subunit of subcomplex B 1, chloroplastic	4.43
PNSB2	Q94AQ8	Photosynthetic NDH subunit of subcomplex B 2, chloroplastic	5.11
PNSB3	Q9LU21	Photosynthetic NDH subunit of subcomplex B 3, chloroplastic	3.64
PNSB4	Q8RXS1	Photosynthetic NDH subunit of subcomplex B 4, chloroplastic	3.62
PNSB5	Q9FG89	Photosynthetic NDH subunit of subcomplex B 5, chloroplastic	4.93
PETE	P00299	Plastocyanin A, chloroplastic	3.40
At2g21870	Q9SJ12	Probable ATP synthase 24 kDa subunit, mitochondrial	2.77
At4g27270	Q6NQE2	Probable NAD(P)H dehydrogenase (quinone) FQR1-like 1	6.99
STR4	Q9M158	Rhodanese-like domain-containing protein 4, chloroplastic	4.61
STR9	O48529	Rhodanese-like domain-containing protein 9, chloroplastic	8.75

Table S8. Fifty-eight genes associated with secondary metabolism at NaCl (300 mmol/L) vs. CK.

Gene name	SwissProt ID	Protein name	log_2FC (NaCl vs. CK)
MENB	Q8GYN9	1,4-dihydroxy-2-naphthoyl-CoA synthase, peroxisomal	4.58
ACO1	Q9MB94	1-aminocyclopropane-1-carboxylate oxidase	3.67
ACO3	Q08507	1-aminocyclopropane-1-carboxylate oxidase 3	9.11
IN37	P23525	2-methyl-6-phytyl-1,4-hydroquinone methyltransferase, chloroplastic	4.36
2ODD19	Q6Z244	2-oxoglutarate-dependent dioxygenase 19	4.35
AOP2	Q945B5	2-oxoglutarate-dependent dioxygenase AOP2	-1.11
UGT85A23	F8WLS6	7-deoxyloganetin glucosyltransferase	3.88
ADH2	Q96533	Alcohol dehydrogenase class-3	3.45
CYP707A2	O81077	Abscisic acid 8'-hydroxylase 2	2.35
CYP707A2	K4CI52	Abscisic acid 8'-hydroxylase CYP707A2	4.05
AAO3	Q7G9P4	Abscisic-aldehyde oxidase	-2.50
RhGT1	Q4R1I9	Anthocyanidin 5,3-O-glucosyltransferase	3.19
COMT1	Q43609	Caffeic acid 3-O-methyltransferase	4.61
CAMT	Q43095	Caffeoyl-CoA O-methyltransferase	9.14
CCD1	Q8LP17	Carotenoid 9,10(9',10')-cleavage dioxygenase 1	3.60
CHS2	P48387	Chalcone synthase 2	-1.31
CCR1	Q9S9N9	Cinnamoyl-CoA reductase 1	3.91
CAD1	Q2KNL5	Cinnamyl alcohol dehydrogenase 1	8.90
SMT1	Q9LM02	Cycloartenol-C-24-methyltransferase	-9.90
CYP72A15	Q9LUC5	Cytochrome P450 72A15	-2.81
CYP72A225	W8JWW3	Cytochrome P450 72A225	-1.23
CYP736A117	A0A068Q6L2	Cytochrome P450 736A117	1.75
CYP76C1	O64636	Cytochrome P450 76C1	4.90
CYP81Q32	W8JMU7	Cytochrome P450 81Q32	-4.07
CYP82A3	O49858	Cytochrome P450 82A3	3.31
CYP84A1	Q42600	Cytochrome P450 84A1	-2.19
CKX6	Q9LY71	Cytokinin dehydrogenase 6	1.13
SOT15	Q8L5A7	Cytosolic sulfotransferase 15	4.27
SC5D	Q9ZT29	Delta(7)-sterol-C5(6)-desaturase	8.81
DHCR24	Q15392	Delta(24)-sterol reductase	5.01
GALUR	O49133	D-galacturonate reductase	2.10
FDH1	Q07511	Formate dehydrogenase, mitochondrial	9.66
VTC2	Q8RWE8	GDP-L-galactose phosphorylase 1	4.17
MIOX4	Q8H1S0	Inositol oxygenase 4	-1.55
LAC14	Q9FY79	Laccase-14	10.59
TT10	Q84J37	Laccase-15	-1.08
GULLO3	Q9LYD8	L-gulonolactone oxidase 3	-1.25
MSBP2	Q9M2Z4	Membrane steroid-binding protein 2	3.49
MYRS	Q93X23	Myrcene synthase, chloroplastic	8.62
PAL1	O64963	Phenylalanine ammonia-lyase 1	4.95
BETV6	Q9FUW6	Phenylcoumaran benzylic ether reductase Betv6	4.70
THIC	O82392	Phosphomethylpyrimidine synthase, chloroplastic	3.71
YUC10	Q9FVQ0	Probable indole-3-pyruvate monooxygenase YUCCA10	1.66
At1g67280	Q8W593	Probable lactoylglutathione lyase, chloroplastic	2.95
PAP3	O82291	Probable plastid-lipid-associated protein 3, chloroplastic	9.24
LGU	Q39366	Putative lactoylglutathione lyase	3.56
SILD	Q94KL7	Secoisolariciresinol dehydrogenase	10.63
SFGH	Q8LAS8	S-formylglutathione hydrolase	2.78
SQE3	Q8VYH2	Squalene epoxidase 3	5.82
THI1-2	F6H7K5	Thiamine thiazole synthase 2, chloroplastic	-1.02
THI1	O23787	Thiamine thiazole synthase, chloroplastic	3.66
CYP73A16	Q43054	Trans-cinnamate 4-monooxygenase	5.77
RHM1	Q9SYM5	Trifunctional UDP-glucose 4,6-dehydratase/UDP-4-keto-6-deoxy-D-glucose 3,5-epimerase/UDP-4-keto-L-rhamnose-reductase RHM1	5.82
CYP92C6	A0A1D6F9Y9	Trimethyltridecatetraene synthase	1.07
UGT89B2	Q6VAA5	UDP-glycosyltransferase 89B2	-2.25
HDLBP	Q00341	Vigilin	8.57
CYP82C4	Q9SZ46	Xanthotoxin 5-hydroxylase CYP82C4	10.38
ABA2	O81360	Zeaxanthin epoxidase, chloroplastic	4.08

Table S9. Twenty-one genes associated with polynucleotide metabolism at NaCl (300 mmol/L) vs. CK.

Gene name	SwissProt ID	Protein name	log_2FC (NaCl vs. CK)
ACIN1	Q9UKV3	Apoptotic chromatin condensation inducer in the nucleus	3.62
FAS2	Q6ZD63	Chromatin assembly factor 1 subunit FAS2 homolog	-1.07
DRT100	Q00874	DNA damage-repair/toleration protein DRT100	4.25
SSRP1	O04235	FACT complex subunit SSRP1	5.37
At5g07800	Q9FF12	Flavin-containing monooxygenase FMO GS-OX-like 9	-1.36
GRP1A	P49310	Glycine-rich RNA-binding protein GRP1A	4.09
HDT2	Q9M4U5	Histone deacetylase HDT2	4.98
H1	P40267	Histone H1	4.52
H2A	P19177	Histone H2A	9.40
H2B-3	O65819	Histone H2B.3	4.74
HTR4	P59169	Histone H3.3	4.67
HMG1	P26585	HMG1/2-like protein	4.18
LARP6A	Q94A38	La-related protein 6A	0.98
LARP6B	O80567	La-related protein 6B	3.57
NDK1	P39207	Nucleoside diphosphate kinase 1	3.82
NAP1;2	Q9ZUP3	Nucleosome assembly protein 1;2	4.71
MRL1	Q0WLC6	Pentatricopeptide repeat-containing protein MRL1, chloroplastic	3.91
PTAC10	F4JF21	Protein PLASTID TRANSCRIPTIONALLY ACTIVE 10	5.45
R3HDM2	A0JNC2	R3H domain-containing protein 2	4.01
ECT2	Q9LJE5	YTH domain-containing protein ECT2	4.48
ECT4	A0A1P8AS03	YTH domain-containing protein ECT4	5.54

Table S10. Two hundred and eighty-six genes associated with translation at NaCl (300 mmol/L) vs. CK.

Gene name	SwissProt ID	Protein name	log_2FC (NaCl vs. CK)
CPN10-1	Q9M1C2	10 kDa chaperonin 1, chloroplastic	5.56
PSRP2	P82277	30S ribosomal protein 2, chloroplastic	5.69
At1g68590	Q9SX22	30S ribosomal protein 3-1, chloroplastic	4.26
RPS1	P29344	30S ribosomal protein S1, chloroplastic	4.00
RPS10	Q9LK61	30S ribosomal protein S10, chloroplastic	3.96
RPS13	P42732	30S ribosomal protein S13, chloroplastic	4.64
RPS17	P16180	30S ribosomal protein S17, chloroplastic	5.60
rps21	P82024	30S ribosomal protein S21, chloroplastic	4.55
RPS31	O80439	30S ribosomal protein S31, chloroplastic	5.16
rps5	P93014	30S ribosomal protein S5, chloroplastic	4.81
RPS6	Q8VY91	30S ribosomal protein S6 alpha, chloroplastic	3.51
RPS9	Q9XJ27	30S ribosomal protein S9, chloroplastic	3.61
ROC4	P19683	31 kDa ribonucleoprotein, chloroplastic	3.29
RPS0	B2B1Z3	40S ribosomal protein S0	-11.08
rps10	O77082	40S ribosomal protein S10	-10.49
RPS11	Q3T0V4	40S ribosomal protein S11	-0.21
RPS12	Q9SMI3	40S ribosomal protein S12	-4.32
RPS13	P62302	40S ribosomal protein S13	-3.38
RS142	P19951	40S ribosomal protein S14	-11.12
RPS14C	P42036	40S ribosomal protein S14-3	3.87
RPS15	P34737	40S ribosomal protein S15	-10.94
RPS15A	Q08112	40S ribosomal protein S15-1	5.48
RPS15A	Q76I82	40S ribosomal protein S15a	-10.78
RPS16	P46293	40S ribosomal protein S16	3.85
RPS17	P08708	40S ribosomal protein S17	4.75
RPS18	Q3T0R1	40S ribosomal protein S18	4.26
RPS19A	Q9SGA6	40S ribosomal protein S19-1	3.34
rps1901	P58234	40S ribosomal protein S19-A	-10.84
RPS2	P15880	40S ribosomal protein S2	4.22
rps20	P23403	40S ribosomal protein S20	-11.30
RPS20B	Q9STY6	40S ribosomal protein S20-2	5.24
RPS23	Q3T199	40S ribosomal protein S23	9.80
RPS23A	P0CY39	40S ribosomal protein S23-A	-10.57
RPS2D	Q9SCM3	40S ribosomal protein S2-4	4.64
RPS24A	Q9SS17	40S ribosomal protein S24-1	-2.56
rps25	Q6PBI5	40S ribosomal protein S25	-11.32
rps2602	Q9UTG4	40S ribosomal protein S26-B	-10.88

RPS27B	Q9M2F1	40S ribosomal protein S27-2	10.41
RPS3	Q3T169	40S ribosomal protein S3	3.22
RPS3B	Q9M339	40S ribosomal protein S3-2	-11.08
RPS3C	Q9FJA6	40S ribosomal protein S3-3	3.02
RS3A	Q8GTE3	40S ribosomal protein S3a	4.58
RPS4	O22424	40S ribosomal protein S4	3.87
RPS4X	Q76N24	40S ribosomal protein S4, X isoform	3.79
RPS5	O65731	40S ribosomal protein S5	-11.00
RPS6	P62753	40S ribosomal protein S6	5.89
RPS6B	P51430	40S ribosomal protein S6-2	3.97
RPS7	Q8LJU5	40S ribosomal protein S7	3.17
RPS8	P49199	40S ribosomal protein S8	9.15
RPS9B	Q9LXG1	40S ribosomal protein S9-1	-11.08
RPS9C	Q9FLF0	40S ribosomal protein S9-2	10.03
RAP40	O65751	40S ribosomal protein SA	4.41
RPL1	Q9LY66	50S ribosomal protein L1, chloroplastic	2.86
RPL10	Q9FY50	50S ribosomal protein L10, chloroplastic	3.86
RPL11	Q9MAP3	50S ribosomal protein L11, chloroplastic	4.48
RK12	P36688	50S ribosomal protein L12, chloroplastic	4.06
RPL13	Q9SYL9	50S ribosomal protein L13, chloroplastic	3.19
RPL15	P25873	50S ribosomal protein L15, chloroplastic	4.30
RPL17	O80363	50S ribosomal protein L17, chloroplastic	3.35
RPL18	Q9SX68	50S ribosomal protein L18, chloroplastic	4.61
At4g17560	Q8W463	50S ribosomal protein L19-1, chloroplastic	3.93
RPL21	P51412	50S ribosomal protein L21, chloroplastic	3.39
RPL24	P92959	50S ribosomal protein L24, chloroplastic	4.40
RPL27	P82190	50S ribosomal protein L27, chloroplastic	3.39
RPL3	O80360	50S ribosomal protein L3, chloroplastic	3.86
RPL31	Q9FWS4	50S ribosomal protein L31, chloroplastic	4.11
RPL35	P23326	50S ribosomal protein L35, chloroplastic	9.63
RPL4	O50061	50S ribosomal protein L4, chloroplastic	4.09
RPL5	O04603	50S ribosomal protein L5, chloroplastic	4.31
RPL6	O23049	50S ribosomal protein L6, chloroplastic	2.96
RPL9	P25864	50S ribosomal protein L9, chloroplastic	4.23
RP-P0	O24573	60S acidic ribosomal protein P0	-11.32
RPP0B	Q42112	60S acidic ribosomal protein P0-2	4.65
RPP1B	O23095	60S acidic ribosomal protein P1-2	5.30
RPL10	P27635	60S ribosomal protein L10	4.25
SC34	A2ZCQ7	60S ribosomal protein L10-1	-11.03
rpl10a	Q6PC69	60S ribosomal protein L10a	-11.08
RPL11	P46287	60S ribosomal protein L11	3.73
RPL11A	P42795	60S ribosomal protein L11-1	-10.29
RPL12	P61284	60S ribosomal protein L12	10.54
rpl1201	P0CT83	60S ribosomal protein L12-A	-10.27
RPL13	P26373	60S ribosomal protein L13	4.00
RL13A	P93099	60S ribosomal protein L13a	-10.98
RPL14	Q3T0U2	60S ribosomal protein L14	-9.93
RPL14A	Q9SIM4	60S ribosomal protein L14-1	5.46
RPL15	O82528	60S ribosomal protein L15	3.17
SB62	O65082	60S ribosomal protein L15-2	-10.26
RPL17B	P51413	60S ribosomal protein L17-2	-3.27
RPL18B	P42791	60S ribosomal protein L18-2	-3.68
RPL18A	Q9ATF5	60S ribosomal protein L18a	1.27
RPL18AA	Q8L7K0	60S ribosomal protein L18a-1	-11.08
RPL19C	P49693	60S ribosomal protein L19-3	4.29
RPL21	O82574	60S ribosomal protein L21	-10.64
RPL21E	Q9FDZ9	60S ribosomal protein L21-2	3.43
Rpl22l1	Q9D7S7	60S ribosomal protein L22-like 1	-5.12
RPL23A	P49690	60S ribosomal protein L23	3.10
RPL23A	O22644	60S ribosomal protein L23A	9.63
RPL24	Q862I1	60S ribosomal protein L24	9.54
RPL24A	Q42347	60S ribosomal protein L24-1	4.07
RPL26	P61257	60S ribosomal protein L26	10.64
RPL26A	P51414	60S ribosomal protein L26-1	4.36
RPL27	P0DJ19	60S ribosomal protein L27	-10.54
RPL27A	P46776	60S ribosomal protein L27a	10.85
RPL27AC	P49637	60S ribosomal protein L27a-3	9.98

RPL28A	O82204	60S ribosomal protein L28-1	3.81
RPL28C	Q9M0E2	60S ribosomal protein L28-2	-9.75
RPL29	P47914	60S ribosomal protein L29	3.71
RPL3	P39023	60S ribosomal protein L3	5.24
RPL31	Q9M573	60S ribosomal protein L31	4.17
RPL32	E2RKA8	60S ribosomal protein L32	-10.34
ARP2	P22738	60S ribosomal protein L3-2	3.64
RPL32A	P49211	60S ribosomal protein L32-1	3.88
RPL34	P40590	60S ribosomal protein L34	3.23
Rpl35	Q6ZWV7	60S ribosomal protein L35	-10.81
RPL35AC	Q9C912	60S ribosomal protein L35a-3	-10.64
RPL36C	Q9LZ57	60S ribosomal protein L36-3	-10.25
rpl3702	P05733	60S ribosomal protein L37-B	-10.44
RPL4	P36578	60S ribosomal protein L4	6.51
RPL5	Q6UNT2	60S ribosomal protein L5	5.24
RPL5B	P49227	60S ribosomal protein L5-2	3.11
RPL6	Q02878	60S ribosomal protein L6	6.03
RPL7	P18124	60S ribosomal protein L7	4.83
RPL7D	Q9LHP1	60S ribosomal protein L7-4	6.03
RPL7A	P62424	60S ribosomal protein L7a	5.24
RPL7A-1	P35685	60S ribosomal protein L7a-1	4.04
RPL8	Q3T0S6	60S ribosomal protein L8	4.92
RPL8C	Q42064	60S ribosomal protein L8-3	3.53
RPL9	P30707	60S ribosomal protein L9	3.46
POPTRDRAFT_821063	B9HQZ6	Alanine--tRNA ligase, chloroplastic/mitochondrial	7.16
EMB1027	O23247	Arginine--tRNA ligase, chloroplastic/mitochondrial	3.18
IBI1	Q9M084	Aspartate--tRNA ligase 2, cytoplasmic	8.64
BAG5	O65373	BAG family molecular chaperone regulator 5, mitochondrial	-1.38
BBD2	Q93VH2	Bifunctional nuclease 2	3.72
BT1	Q9FMK7	BTB/POZ and TAZ domain-containing protein 1	2.56
At1g63850	Q9CAJ9	BTB/POZ domain-containing protein At1g63850	8.68
CALR	O81919	Calreticulin	3.91
Cpe	Q00493	Carboxypeptidase E	2.13
CPN60B2	Q9LJE4	Chaperonin 60 subunit beta 2, chloroplastic	3.92
CSP41A	Q9LYA9	Chloroplast stem-loop binding protein of 41 kDa a, chloroplastic	4.82
CSP41B	Q9SA52	Chloroplast stem-loop binding protein of 41 kDa b, chloroplastic	4.08
CUL1	Q5ZC88	Cullin-1	8.86
RH15	Q56XG6	DEAD-box ATP-dependent RNA helicase 15	4.13
RH3	Q8L7S8	DEAD-box ATP-dependent RNA helicase 3, chloroplastic	3.60
Os01g0911100	Q5N7W4	DEAD-box ATP-dependent RNA helicase 30	5.64
RH31	Q9FFQ1	DEAD-box ATP-dependent RNA helicase 31	3.17
RH50	Q8GUG7	DEAD-box ATP-dependent RNA helicase 50	8.71
RH53	Q9LUW5	DEAD-box ATP-dependent RNA helicase 53, mitochondrial	2.94
RH7	Q39189	DEAD-box ATP-dependent RNA helicase 7	4.38
DESI1	Q6ICB0	Desumoylating isopeptidase 1	3.53
dnajb6	Q6P642	DnaJ homolog subfamily B member 6	4.70
OST48	Q944K2	Dolichyl-diphosphooligosaccharide--protein glycosyltransferase 48 kDa subunit	4.87
STT3B	Q9FX21	Dolichyl-diphosphooligosaccharide--protein glycosyltransferase subunit STT3B	4.27
RZPF34	Q9FFB6	E3 ubiquitin-protein ligase RZFP34	1.72
UPL1	Q8GY23	E3 ubiquitin-protein ligase UPL1	3.80
REFA1	O64937	Elongation factor 1-alpha	6.74
EEF1A1	P68103	Elongation factor 1-alpha 1	5.29
At5g12110	Q84WM9	Elongation factor 1-beta 1	4.86
EEF1G	P26641	Elongation factor 1-gamma	4.56
Os02g0220500	Q6YW46	Elongation factor 1-gamma 2	4.02
EEF2	P13639	Elongation factor 2	5.31
fusA1	P34811	Elongation factor G-1, chloroplastic	3.75
TUFB1	P46280	Elongation factor Tu, chloroplastic	4.35
HSPA5	P11021	Endoplasmic reticulum chaperone BiP	5.73
HSP90B1	P14625	Endoplasmin	4.82
HSP90	P35016	Endoplasmin homolog	4.08
IF4A8	P41381	Eukaryotic initiation factor 4A-8	3.78
EIF4A1	Q3SZ54	Eukaryotic initiation factor 4A-I	3.91
EIF4A3A	Q5VNM3	Eukaryotic initiation factor 4A-III homolog A	4.87
Gspt1	Q8R050	Eukaryotic peptide chain release factor GTP-binding subunit ERF3A	8.53

ERF1-3	P35614	Eukaryotic peptide chain release factor subunit 1-3	3.93
IF1A	P56331	Eukaryotic translation initiation factor 1A	4.65
TIF3B1	P56821	Eukaryotic translation initiation factor 3 subunit B	7.26
TIF3C1	Q9XHM1	Eukaryotic translation initiation factor 3 subunit C	3.81
TIF3E1	Q9C5Z3	Eukaryotic translation initiation factor 3 subunit E	4.94
TIF3F1	O04202	Eukaryotic translation initiation factor 3 subunit F	8.75
EIF4G2	Q95L46	Eukaryotic translation initiation factor 4 gamma 2	3.29
EIF4B	P23588	Eukaryotic translation initiation factor 4B	4.91
EIF4B1	Q9LIN5	Eukaryotic translation initiation factor 4B1	8.76
IF5A	Q9AXJ4	Eukaryotic translation initiation factor 5A	4.58
EIF-5A2	P24922	Eukaryotic translation initiation factor 5A-2	3.23
EIF(ISO)4G1	Q93ZT6	Eukaryotic translation initiation factor isoform 4G-1	4.52
At1g29880	O23627	Glycine--tRNA ligase, mitochondrial 1	4.91
RNP1	Q8W034	Heterogeneous nuclear ribonucleoprotein 1	3.72
HNRNPA1	P09867	Heterogeneous nuclear ribonucleoprotein A1	4.23
LONP2	O04979	Lon protease homolog 2, peroxisomal	3.82
BIP4	Q03684	Luminal-binding protein 4	5.23
BIP5	Q03685	Luminal-binding protein 5	-1.17
MRF1	Q94BR1	MA3 DOMAIN-CONTAINING TRANSLATION REGULATORY FACTOR 1	4.32
MORF5	Q9C7Y2	Multiple organellar RNA editing factor 5, chloroplastic/mitochondrial	3.14
MORF8	Q9LKA5	Multiple organellar RNA editing factor 8, chloroplastic/mitochondrial	4.33
MORF9	Q9LPZ1	Multiple organellar RNA editing factor 9, chloroplastic	3.21
RCE1	Q9SDY5	NEDD8-conjugating enzyme Ubc12	4.08
PAPS2	O82312	Nuclear poly(A) polymerase 2	8.89
PAPS4	Q8VYW1	Nuclear poly(A) polymerase 4	8.77
Os04g0620700	Q7XTT4	Nucleolin 2	3.13
UBP1	Q9M427	Oligouridylate-binding protein 1	4.32
At5g46580	Q9LS25	Pentatricopeptide repeat-containing protein At5g46580, chloroplastic	3.51
PNAA	P81898	Peptide-N4-(N-acetyl-beta-glucosaminyl)asparagine amidase A	3.39
PCKR1	Q39613	Peptidyl-prolyl cis-trans isomerase	3.74
PPIA	P62938	Peptidyl-prolyl cis-trans isomerase A	3.83
PPIB	P23284	Peptidyl-prolyl cis-trans isomerase B	9.52
CYP20-3	P34791	Peptidyl-prolyl cis-trans isomerase CYP20-3, chloroplastic	4.88
CYP28	O65220	Peptidyl-prolyl cis-trans isomerase CYP28, chloroplastic	9.02
CYP40	Q9C566	Peptidyl-prolyl cis-trans isomerase CYP40	1.12
CYP95	Q8RWY7	Peptidyl-prolyl cis-trans isomerase CYP95	3.55
FKBP18	Q9LM71	Peptidyl-prolyl cis-trans isomerase FKBP18, chloroplastic	8.68
FKBP65	Q9FJL3	Peptidyl-prolyl cis-trans isomerase FKBP65	3.55
PABPC1	P61286	Polyadenylate-binding protein 1	3.38
pabpc1a	F1QB54	Polyadenylate-binding protein 1A	-9.88
PAB2	P42731	Polyadenylate-binding protein 2	4.99
PAB8	Q9FXA2	Polyadenylate-binding protein 8	3.44
RBP45	Q9LEB4	Polyadenylate-binding protein RBP45	4.53
RBP47	Q9LEB3	Polyadenylate-binding protein RBP47	3.95
CID7	O64843	Polyadenylate-binding protein-interacting protein 7	4.25
PETs	Q9SZD6	Polyprotein of EF-Ts, chloroplastic	3.90
PCUBI4-1	P0CH04	Polyubiquitin	5.18
UBB	P0CG55	Polyubiquitin-B	9.29
UBC	P0CG68	Polyubiquitin-C	3.56
ubqD	P0CG77	Polyubiquitin-D	4.11
PRP8A	Q9SSD2	Pre-mRNA-processing-splicing factor 8A	8.86
At1g65660	Q9SHY8	Pre-mRNA-splicing factor SLU7-A	4.75
PREP2	Q8VY06	Presequence protease 2, chloroplastic/mitochondrial	4.89
RZFP34	Q5JL96	Probable E3 ubiquitin-protein ligase RZFP34	5.16
At4g00750	Q9ZPH9	Probable methyltransferase PMT15	-4.60
At1g26850	B9DFI7	Probable methyltransferase PMT2	5.51
At1g31850	Q9C6S7	Probable methyltransferase PMT20	4.34
At1g04430	Q940J9	Probable methyltransferase PMT8	3.97
UBC24	Q8VY10	Probable ubiquitin-conjugating enzyme E2 24	5.37
SEC	Q9M8Y0	Probable UDP-N-acetylglucosamine--peptide N-acetylglucosaminyltransferase SEC	4.23
AGO1	O04379	Protein argonaute 1	3.81
AGO2	Q9SHF3	Protein argonaute 2	4.67
AGO4	Q9ZVD5	Protein argonaute 4	3.79
BSD2	Q9SN73	Protein BUNDLE SHEATH DEFECTIVE 2, chloroplastic	3.97
DCP5	Q9C658	Protein decapping 5	4.42

PDIL1-4	Q9FF55	Protein disulfide isomerase-like 1-4	4.58
P4HB	P07237	Protein disulfide-isomerase	6.06
PDIA3	P30101	Protein disulfide-isomerase A3	4.35
PDIA6	Q15084	Protein disulfide-isomerase A6	4.14
grpE	Q8DJB3	Protein GrpE	8.82
VIP4	Q9FNQ0	Protein LEO1 homolog	8.58
NCA1	Q9M2V1	Protein NCA1	3.63
TAR1-A	Q6CQE5	Protein TAR1	-10.79
GOS2	A6MZM2	Protein translation factor SUI1 homolog	4.50
SVR3	F4K410	Putative elongation factor TypA-like SVR3, chloroplastic	5.38
DDB_G0268948	Q55EX9	Putative methyltransferase DDB_G0268948	9.58
RNC1	Q9SZV0	Ribonuclease III domain-containing protein RNC1, chloroplastic	3.98
PSRP1	P19954	Ribosome-binding factor PSRP1, chloroplastic	3.56
RIPt	Q9M653	Ribosome-inactivating protein PMRIPt	4.73
RRF	P37706	Ribosome-recycling factor, chloroplastic	3.28
RBCS	P24007	Ribulose bisphosphate carboxylase small subunit, chloroplastic	3.53
RCA2	Q7X999	Ribulose bisphosphate carboxylase/oxygenase activase 2, chloroplastic	3.71
RPE	Q43843	Ribulose-phosphate 3-epimerase, chloroplastic	3.43
ATL2	Q8L9T5	RING-H2 finger protein ATL2	-1.72
ATL32	Q8W571	RING-H2 finger protein ATL32	1.34
RBM39	Q5RC80	RNA-binding protein 39	9.13
CP29B	Q9ZUU4	RNA-binding protein CP29B, chloroplastic	4.59
RUBA	P08926	RuBisCO large subunit-binding protein subunit alpha, chloroplastic	3.88
SAMT	Q9SPV4	Salicylate carboxymethyltransferase	5.94
RS41	P92966	Serine/arginine-rich splicing factor RS41	3.85
SR30	Q9XFR5	Serine/arginine-rich splicing factor SR30	8.96
FFC	P37107	Signal recognition particle 54 kDa protein, chloroplastic	3.16
SKP1A	Q39255	SKP1-like protein 1A	10.09
SUMO1	P55852	Small ubiquitin-related modifier 1	3.45
SPP	Q9FIH8	Stromal processing peptidase, chloroplastic	3.65
At2g25737	Q8L7A0	Sulfite exporter TauE/SafE family protein 3	4.67
CCT3	P49368	T-complex protein 1 subunit gamma	8.34
YLS8	Q9FE62	Thioredoxin-like protein YLS8	3.73
THRRS	O04630	Threonine--tRNA ligase, mitochondrial 1	5.25
At1g17220	Q9SHI1	Translation initiation factor IF-2, chloroplastic	3.48
IF3-2	O82234	Translation initiation factor IF3-2, chloroplastic	4.17
TRAM1	Q15629	Translocating chain-associated membrane protein 1	6.03
TIG	Q8S9L5	Trigger factor-like protein TIG, Chloroplastic	4.83
RNU1	Q42404	U1 small nuclear ribonucleoprotein 70 kDa	9.51
UBP23	Q9FPS4	Ubiquitin carboxyl-terminal hydrolase 23	3.51
UCHL1	P09936	Ubiquitin carboxyl-terminal hydrolase isozyme L1	4.91
UBP13	Q84WU2	Ubiquitin C-terminal hydrolase 13	4.01
DSK2B	Q9SII8	Ubiquitin domain-containing protein DSK2b	4.26
RL40	P46575	Ubiquitin-60S ribosomal protein L40	-11.41
UBA1	P93028	Ubiquitin-activating enzyme E1 1	5.11
UBC28	Q94F47	Ubiquitin-conjugating enzyme E2 28	3.65
UBC35	Q94A97	Ubiquitin-conjugating enzyme E2 35	4.36
N/A	P35135	Ubiquitin-conjugating enzyme E2-17 kDa	4.63
UBC4	P16577	Ubiquitin-conjugating enzyme E2-23 kDa	8.98
UBL5	Q9FGZ9	Ubiquitin-like protein 5	10.73
XRCC6	P12956	X-ray repair cross-complementing protein 6	8.04
YBX1	P67808	Y-box-binding protein 1	2.46

Table S11. Sequences of primer used in qRT-PCR validation.

Genes	Sequences (5' to 3')	Amplicon size (bp)
ACT	Forward: TGGTGTTAGCCACACAGTCC	159
	Reverse: CTCACAATTTCACGCTCGGC	
**Salt stress (10)**		
ALDH7B4	Forward: ACCAGGTGCGATCTTTACGG	
	Reverse: ACCATTTGACCCACCTTCGT	117
ANN2	Forward: TTGGTTCCCCTTGTGAGTGC	
	Reverse: CCTCCTCGTCACTGTAAGCC	121
RD22	Forward: CAATATGCAGCAACCGCGAC	153
	Reverse: CGTTTTGGCAACTTTGCGTG	
CAMBP25	Forward: CTGAAACGCTCACCAAAGCC	117

	Reverse: TCGAAACGGGGTCAGAAGTG	
PCC13	Forward: GGGAAGAATTTGCAGCCTCC<br>Reverse: TCTTGACCGGATTCCACACC	176
RBG7	Forward: TTTAGGTGTTTCGTTGGCGG<br>Reverse: TCATCGACTTCTCATCGCCG	166
IQM4	Forward: GAGGAACGTCCTACAGCGTC<br>Reverse: AAGCATCCCTGTTTGTCGGT	112
NFD4	Forward: GGTCGAGTGACCAAACGGAT<br>Reverse: CCGCTTCTTCCTCTACCGTC	108
WSD1	Forward: ATGCCACGGATTTTGTCTGG<br>Reverse: GTTGTTACCCTGACTGGCCG	143
At2g40140	Forward: AGTCACATGGCTCTGATGGC<br>Reverse: TGCTCAAAGACCTCACGCAT	174
**Antioxidant enzyme activities (12)**		
SODCP	Forward: CTCCCTCAACGCCAGAACTT<br>Reverse: CATCGTCCTCCTGGGTCAAC	142
FSD2	Forward: TGCGACAAATCCCCTTCCAT<br>Reverse: TGTAATCGGGCCTTCGGTTC	161
SODA	Forward: TCTCGCTCCAGTTCGTGAAG<br>Reverse: CTGCACCTTCCGCATTCATC	119
At5g06290	Forward: CTTCAGTCTCGGGTTCCTCG<br>Reverse: TCGAAAACGGCTTCTGCTTC	137
APX3	Forward: AAGAGATCGACAAGGCTCGC<br>Reverse: TCGCACCATGACTAAGCTCC	171
poxN1	Forward: TCCAACGATTCTTCGCCCTT<br>Reverse: ACGAATTTCACCTTGCGTGC	104
PRDX1	Forward: TGTTGGGACTGCTGATAGGAAGATG<br>Reverse: GGGGCACACAAAGGTGAAGTC	181
PEX11C	Forward: GCTTGGAAGGTCAGGCATCT<br>Reverse: CCAGCTCTCCAAGCTCAACT	119
GPX1	Forward: CCCGCGGTCAAAAACACAC<br>Reverse: AAACACCGAAGGGGAGACCT	179
CATA	Forward: GGAGCCAGTGCTAAGGGTTT<br>Reverse: AGAACCGAACGATGAGAGGC	109
CAT1	Forward: CCAGAACGTGTTGTCCATGC<br>Reverse: AGGATCCCTCAGCGTTTCAG	180
PNC1	Forward: TTGCTGCTCGTGAATCCGTT<br>Reverse: GAAGTCCCAAAACGGGGGAG	128
**Soluble sugar and protein metabolism (23)**		
G6PDC	Forward: CCGAAGGATAGTCTCACGCC<br>Reverse: AGAGCTTTGCCTGCCTTCAT	101
GAPA1	Forward: CGCTGATGTCAAGCCTGTTG<br>Reverse: CTCCATCCCTGTCCACGAAC	155
PGMP	Forward: GGGAGGTGATGGCCGTTATT<br>Reverse: CCCACCATTGGCCTTTCTCT	157
UGPA	Forward: CTTGTGGAGGCTGATGCACT<br>Reverse: TGGGAGAAATCGGGAACGAG	156
INVE	Forward: GCGTGTTGCACCTGTTGATT<br>Reverse: GTCAAAACCATCAGCGAGGC	151
SUS2	Forward: AGAGAGGTTGGGGAGACACT<br>Reverse: AGAATCCGTGCGGAGACAAG	153
FKFBP	Forward: TTCTCAGCCGAAGCCAGATG<br>Reverse: TGGCGATCCTCTTTTGGTCC	198
F16P2	Forward: CCTGCACGCTTGTGTTAAGC<br>Reverse: GGTTGTTGGAGCATCCCAGT	164
FBA3	Forward: CTGTGGGAAGAGATTGGCGT<br>Reverse: GCGCAAGCAGTCAACAAACT	178
GOLS1	Forward: AAAGGCGTTGTTGGATTGGC<br>Reverse: ACGGGTTCGATTTCCCTCAC	143
TPS6	Forward: AGTCGGCAAGGAAACGAGAG<br>Reverse: ACGGGGAGCAACCAATGAAT	103
OFUT39	Forward: GCATTGGGCTTCGACAACAG<br>Reverse: GTCAAGTGCGGCTAGCAAAG	180
DPE2	Forward: AGGTGTGGGTGTAGCAATCC	168

	Reverse: TCCCACCACATCCCATGAAC	
AMY3	Forward: ATATTGGCGGCTGTCGGATT<br>Reverse: TGACCCTGAGTAGAGCCAGT	111
BAM1	Forward: GGGTGAGCTTCGTTACCCTT<br>Reverse: GTGCTACCCCATTCTGGCTT	147
GWD3	Forward: ATAAAGGTGGGGCTCGTTGG<br>Reverse: AAGACCCTTCTCTTGCCACG	176
RPN1A	Forward: TGGAAAAACAAGTGCGGCAG<br>Reverse: GCCAATGCAGGGTCACAATC	177
PCS1	Forward: TAGGCGACATGGTGTTGGAG<br>Reverse: CCGATCCCTATGCAGTGGAC	103
Cys	Forward: GTGGATCTGCTACACACGGT<br>Reverse: TGGCAACTTGTGGCATTCTTG	105
DEGP1	Forward: TAGTTCTGCGGCTACTGGTC<br>Reverse: TCCAACACCAGAGGATGCAC	160
PBE2	Forward: CATGGTTGCTGCTGATTCTCG<br>Reverse: AACTGAGATCCTGCGCTTGT	193
SBT2.5	Forward: AGCTGGGCTTGGCTTATCAC<br>Reverse: TCTGGTCTCTGACAGTCCGT	123
ALEU	Forward: CCAACAAAAAGGGCCTGTCG<br>Reverse: AGCAATTCTGTGCAGCTCCT	102
**Cell morphogenesis (6)**		
F16	Forward: GATGGTTCTGTGTGTTGCGG<br>Reverse: AGACATGGTGAAGGGACGAC	184
FIP1	Forward: CTGTCCACATCTGCTGGTCC<br>Reverse: ACTTTGCTCGTGGACGGATT	176
At5g13200	Forward: CCCCCGTGGAAAAATCAAGC<br>Reverse: GCTTTTGCCGTCACGTTCAT	166
At5g23350	Forward: AAGAGGAATCCATGGCGAGAG<br>Reverse: TGCTCCAACACTCAGCTTTCC	137
KAI2	Forward: ACTCGGTTTCCGCCATGATT<br>Reverse: CTCCTGTTCAAATCCCCCGT	131
ROH1	Forward: TTCTGGTTCCGACGAGCTTC<br>Reverse: TCACGAATGGCATTGCACAC	195
**Transcription factor (8)**		
MYB73	Forward: TTGAACCCGAGTAGCCCATC<br>Reverse: CCGTCGTCGAAGAAGAGGTC	133
WRKY24	Forward: CGAGTAGCGTTGAAATGCCG<br>Reverse: TCAACGTCCTCTCTTTCGCC	145
WRKY71	Forward: TCTACAACACTAGGCGGTGC<br>Reverse: TGCTGAAACGGGGTTAGGTT	152
NAC083	Forward: ACGAATACCGCCTTGTCAGC<br>Reverse: GCGGCATAAAACCCAACTCT	101
JA2L	Forward: TGGGTTCTTCCCAGCAAAGC<br>Reverse: CAACTTTGCGTCCTTCGGTG	166
BZIP44	Forward: GCACTTGGATGATCTGATGGC<br>Reverse: GAGGTTCATGTAGTGCTGGGT	100
TCP14	Forward: TTCGGATGCCTGCTCTATGC<br>Reverse: GGACATACTGGAGCCGGAAC	197
UNE10	Forward: CGAAAACGACGACAACCACC<br>Reverse: AATAGCGGCAGCTCTACTCC	146
**Hormone response (12)**		
GASA2	Forward: TCTCGTCTCACTTCTGGCTCT<br>Reverse: GCCCTCTTGCATAAGTGTGG	126
ARF2A	Forward: AGTTGGCCTATGATCGCCAC<br>Reverse: TCAACAGATCGTCCAAGGGC	157
IAA9	Forward: GGGCAAGGGAAGTTTTCTGG<br>Reverse: CCGAACGAGACCTTTCCTGT	124
TET4	Forward: GTTTCGTTGGCAGGATTCGC<br>Reverse: CCGACCCCTTTATCCGTCACC	139
CISZOG2	Forward: AAGTGGTTGTGGTCGTCGTG<br>Reverse: TGGCGATTATGGGTTGGAGT	127
AHK3	Forward: ACTGGCATTGGGCTTAGCAT<br>Reverse: ACAAGGAAGAGCTCGGTTGG	175

JOX2	Forward: AGCGTGGAGCATAGGGTAGT<br>Reverse: GTCATGGGTGCGTATTGTGC	143
TIFY6B	Forward: GTGGGTCTTCCATGGCTCTT<br>Reverse: GACCCACTCCCTGACTTTGG	160
PYL3	Forward: GCCGTTTATAAGCAGGTGCG<br>Reverse: AGCCTTTCTGTGCTGGTTGT	105
PP2CA	Forward: AATGGACGGAGAACTCCACG<br>Reverse: TCAGGAGTAACAACGGCGAC	103
ETR2	Forward: TGATACTCCGGGTGGCTTCT<br>Reverse: GTGCCTCCTACCCCCTAGTT	179
ERF013	Forward: TCCTCGCCTTCGTTGTCTTC<br>Reverse: GCCCCATTGAGCATATCGGT	176
**Ion transport (10)**		
CSC1	Forward: AGGTTTACTACCGGGGCTCT<br>Reverse: TAGCTGGTCCAATGCTGTCC	193
CLC-C	Forward: CAGCAGCAGGGTCTGGTATC<br>Reverse: CCCCTGCCCAAGTAAAGAGG	194
RAN1	Forward: TCCCTGTGCTCTTGGATTGG<br>Reverse: TTGACCCGCTGAGCTCTTTC	117
KEA2	Forward: CTGTGGGTTTGGACGTGTTG<br>Reverse: AAGCTCTTTCCGCACCAACT	182
OEP16	Forward: GGTTTCTCGTAGCGGTCTGG<br>Reverse: TCTTTGCCATTGTTGCTCGC	198
PPI1	Forward: TGAGTGCTGATGGACGCATT<br>Reverse: GCCAGTTCAAATTCCCCTGC	151
NHX2	Forward: ATGGGTCTTGTGATGGTCGG<br>Reverse: TATGCCCGGCCCTTGTAAAC	187
CAX1	Forward: CGTGGCGTATGTTGCGTATC<br>Reverse: TACCCACGACGTACTCGGAT	194
VHA-a2	Forward: TCGGTTCATCGTGGCAATCT<br>Reverse: AACCCTCACCGACAAGACAC	145
ZIP1	Forward: GCTGGTGCTATAGGGGTGAG<br>Reverse: GGATTCTGGCTAAGGCAAGGT	182
